# Compositional epistasis detection using a few prototype disease models

**DOI:** 10.1371/journal.pone.0213236

**Published:** 2019-03-27

**Authors:** Lu Cheng, Mu Zhu

**Affiliations:** Department of Statistics and Actuarial Science, University of Waterloo, Waterloo, Ontario, Canada; Auburn University - Harrison School of Pharmacy, UNITED STATES

## Abstract

We study computational approaches for detecting SNP-SNP interactions that are characterized by a set of “two-locus, two-allele, two-phenotype and complete-penetrance” disease models. We argue that existing methods, which use data to determine a best-fitting disease model for each pair of SNPs prior to screening, may be too greedy. We present a less greedy strategy which, for each given pair of SNPs, limits the number of candidate disease models to a set of prototypes determined a priori.

## 1 Introduction

For many years, scientists have tried to identify single-nucleotide polymorphisms (SNPs) that are associated with various diseases, but over the years it is becoming apparent that single genetic variations can explain only very little heritability. This has come to be known as the so-called “missing heritability problem” [1–3], and has prompted many scientists to conjecture that perhaps SNP-SNP interactions are more prevalent than we had previously thought [4].

In genetics, the term “epistasis” refers to the phenomenon that the effect of one gene (or SNP) is dependent on the presence of others. Different definitions of epistasis exist. For example, in biochemical genetics, the term “functional epistasis” is sometimes used to mean the molecular interactions that proteins (and/or other genetic elements) have with one another; whereas in population and/or quantitative genetics, the terms “statistical epistasis” and “compositional epistasis” are often used. The former is due to Fisher [5] and usually taken to mean deviation from additive genetic effects, while the latter emphasizes the notion of having a masking effect—as such, some [6–8] believe it to be closer to the original meaning of the word “epistasis” when Bateson [9] first coined it in 1909. As Phillips [7] wrote, “[c]ompositional epistasis measures the effects of allele substitution against a particular fixed genetic background, while statistical epistasis measures the average effect of allele substitution against the population average genetic background.”

To characterize different compositional epistatic effects, we follow various researchers who have studied this problem and focus on a set of “two-locus, two-allele, two-phenotype, and complete-penetrance” (TTTC) disease models [10]. Table 1 shows a few examples. Often, these disease models can be interpreted as one SNP having a certain masking effect on the other. For instance, the recessive-recessive disease model [Table 1(c)] can be viewed as the major allele “A” from one SNP having a masking effect on the causal genotype “bb” from the other SNP, or as the major allele “B” having a similar masking effect on the causal genotype “aa”.

**Table 1:** Examples of TTTC disease models. A “1” means the corresponding genotype combination, e.g., “aabb” in (c), would elevate the risk of disease, whereas a “0” means it would not.

(a)				(b)			
	AA	Aa	aa		AA	Aa	aa
BB	**1**	**1**	0	BB	0	**1**	0
Bb	**1**	**1**	0	Bb	**1**	0	**1**
bb	0	0	0	bb	0	**1**	0
(c)				(d)			
	AA	Aa	aa		AA	Aa	aa
BB	0	0	0	BB	0	**1**	0
Bb	0	0	0	Bb	**1**	**1**	**1**
bb	0	0	**1**	bb	0	**1**	0

Clearly, these TTTC disease models can describe only two-way interactions between two SNPs, and the notion of epistasis itself certainly does not preclude higher-order interactions among more than two SNPs. At a *genome-wide* level, however, screening for higher-order interactions is still largely impractical. For example, even with 100,000 SNPs, there would be (100,0002)≈5.0×109 or about 5 *billion* SNP-pairs to screen already if we limited ourselves to 2-way interactions only, and (100,0003)≈1.7×1014 SNP-triplets to screen if 3-way interactions were to be considered. Therefore, in this paper, we take a “narrow” point of view by restricting ourselves to consider only two-way interactions.

What’s more, these TTTC models are practically useful, especially when the minor allele frequency (MAF) is low. A TTTC model has two degrees of freedom, corresponding to the two penetrance levels, denoted respectively by “1” and “0” in Table 1; whereas a “full model” will have a 9 degrees of freedom, one for each of the nine genotype combinations. When the MAF is low, there can be insufficient data for some of the rare genotype combinations, making it hard to obtain reliable parameter estimates. (In the extreme case, we may have no data in the sample for a particular genotype combination.) Under such circumstances, it is beneficial to reduce the number of parameters, or the degree of freedom. Using a TTTC model, one only has to estimate two parameters. By limiting the degree of freedom in this way, the power of the statistical test can be improved.

Thus, when we say “epistasis” in this paper, we are largely referring to these TTTC disease models only. Even so, there are still 2^9^ possible TTTC disease models in theory [10] for *each pair* of SNPs, and it is generally not possible to screen them all. But a bad choice of the disease model can be detrimental, in that a pair of SNPs may appear highly associated with an outcome under one disease model and not associated under another. For example, studies on single-locus effects have generally confirmed that the power (of detecting an existing effect) is largest when the correct genetic model—e.g., recessive, dominant, additive, and so on—is specified [11–13], and there is all the reason to expect that the same conclusion will hold for detecting epistatic effects between two SNPs.

Among methods available for choosing a disease model for each pair of SNPs prior to screening, two popular ones are: the multi-factor dimensionality reduction (MDR) method by Ritchie *et al*. [14], and the method by Wan *et al*. [15], which we shall refer to throughout the paper simply as the “ratio split” (RS) method. Both of these methods rely on the case-control ratios of different genotype combinations (i.e., AABB, AABb, and so on) in order to decide on a particular disease model to use for a given pair of SNPs. Specifically, the MDR method determines a disease model by thresholding the case-control ratios; typically, genotype combinations with ratios ≥ 1 (on a balanced case-control sample) are regarded as high risk. The RS method, on the other hand, first sorts the case-control ratios in descending order and evaluates 8 different disease models by sequentially considering the top *x* genotype combinations as high risk, for *x* = 1, 2, …, 8. Then, it chooses the one that best predicts the outcome (e.g., disease).

Both of these methods are essentially greedy and use the data twice: first, to determine the disease model for each pair of SNPs; then, to determine whether each pair of SNPs is associated with the outcome. As such, they can be *overly* adaptive to data, and have a tendency to produce many false positives. The cost of using the data twice is especially pronounced if the sample size is relatively small (which is almost always the case for genome-wide association studies), and/or if the data quality is not so good. Indeed, this kind of concern has been reported in the literature [16], especially in the context of genome-wide studies where extra out-of-sample validation, which can help mitigate such problems, is computationally prohibitive.

Instead of relying on the case-control ratios to determine what disease model to use for a pair of SNPs, our main idea is based on the observation that some of these TTTC disease models are more similar than others. In Table 1, for example, arguably the two models on the left [(a) and (c)] are quite different from, whereas the two on the right [(b) and (d)] are somewhat similar to, each other. While we shall be more specific later (Section 3) about how we propose to measure the similarity between two disease models, such an observation nonetheless means that we can first group all possible disease models into a few clusters, and then select a representative prototype from each cluster for screening purposes. The set of prototype models can be seen to constrain the search space somewhat, in the sense that only disease models in the prototype set are now “permitted”. This allows our method to be less data-adaptive, while including a prototype from each cluster still ensures that we are not systematically missing important parts of the search space. In what follows, we will sometimes use the acronym “PTY” (for “prototype”) to refer to our method, especially in tables and figures.

It is worth mentioning that a cluster analysis of all disease models is beneficial in its own right. For example, it may allow us to better understand and characterize different epistatic effects (more on this below in Section 3.2), for which there have been a few previous endeavours [10, 17–19].

### 1.1 Marginal versus sequential screening

Throughout the paper, we will use the following empirical protocol repeatedly to compare different methods. For any given pair of SNPs, e.g., (*i*, *j*), each method has its own way of determining a “best-fitting” disease model—call it *M*_*i*,*j*_. A *nominal* measure of association between the (*i*, *j*)-pair and the outcome is then computed as the $\chi_{\left(1\right)}^{2}$-statistic for testing whether the risky/non-risky assignment by *M*_*i*,*j*_ is statistically independent of the outcome (i.e., disease or no disease), which we simply denote as ${\hat{\chi }}_{i,j}^{2}$. (We will explain in more detail later in Section 2.3 why we use the adjective “nominal” to describe these association measures.) The pair (*i*, *j*) can then be ranked according to ${\hat{\chi }}_{i,j}^{2}$ or considered having been “selected” or “detected” by the method if ${\hat{\chi }}_{i,j}^{2}$ exceed a certain significance threshold. We refer to this as the “marginal screening procedure”. (One also can use only part of the data to determine *M*_*i*,*j*_, and compute an *out-of-sample* measure of association by testing *M*_*i*,*j*_ against the outcome on the remaining data. For example, MDR is usually applied in this manner when the number of candidate SNPs being studied is relatively small. To reduce variation caused by chance division of the data, however, such a process often needs to be repeated a few times and the resulting measures averaged, thus making it computationally prohibitive for genome-wide screening [20, 21]).

Alternatively, we also can combine the effects of multiple SNP-pairs *sequentially*. For example, after having selected the top pair—call it (*i*_1_, *j*_1_), we can *re-assess* each remaining pair (*i*, *j*) by testing whether the *combined* risky/non-risky assignment by

$$\begin{array}{c} M_{i_{1},j_{1}}\;\text{or}\;M_{i,j} \end{array}$$ (1)

is independent of the outcome. We use ${\hat{\chi }}_{i,j|H}^{2}$ to denote the corresponding test statistic, where H means the entire *history* of pairs already selected so far. (After the top pair has been selected, $H=\{M_{i_{1},j_{1}}\}$; after two pairs have been selected, $H=\{M_{i_{1},j_{1}},M_{i_{2},j_{2}}\}$; and so on.) The pair to be selected next is

$$\begin{array}{c} \underset{M_{i,j}\notin H}{\text{arg}\;\text{max}}\;{\hat{\chi }}_{i,j|H}^{2}, \end{array}$$ (2)

rather than

$$\begin{array}{c} \underset{M_{i,j}\notin H}{\text{arg}\;\text{max}}\;{\hat{\chi }}_{i,j}^{2}. \end{array}$$ (3)

We refer to this as the “sequential screening procedure”.

## 2 Motivation

Before we describe our approach in more detail, we first provide some motivations by discussing some weaknesses of existing methods. We should emphasize that these are merely some *examples* of scenarios in which PTY can be seen to have certain advantages over MDR and RS. They are by no means the only—or even necessarily the main —such scenarios. The reason why they are being presented, rather than others, is because they are still relatively easy for us to describe with a reasonable amount of clarity, whether algebraically (Section 2.1), verbally (Section 2.2), or both (Section 2.3).

### 2.1 A pathological scenario

We begin by considering a pathological scenario. Suppose that two pairs of SNPs (e.g., {A/a, B/b}, {C/c, D/d}) are independent (Table 2). For *i* = 1, 2, …, 9, let *w*_*i*_ be the probability of having the *i*-th genotype combination in the first pair, and likewise *v*_*j*_ for the second pair. For simplicity, suppose each genotype combination is either risky (∈ *R*) or non-risky (∈ *N*). For *k*, *ℓ* ∈ {0, 1}, let *p*_*kℓ*_ be the penetrance level for individuals having risky combinations from both pairs (*k* = *ℓ* = 1), the first pair only (*k* = 1, *ℓ* = 0), the second pair only (*k* = 0, *ℓ* = 1), or neither pair (*k* = *ℓ* = 0). Then, derivations contained in S1 Appendix show that, if

$$\begin{array}{c} p_{11}\sum_{j\in R}v_{j}+p_{10}\sum_{j\in N}v_{j}=p_{01}\sum_{j\in R}v_{j}+p_{00}\sum_{j\in N}v_{j}, \end{array}$$ (4)

the case-control ratio will be the same for all genotype combinations *i* = 1, 2, …, 9 in the first pair, regardless of whether *i* ∈ *R* or *i* ∈ *N*. It is thus a pathological case, in which it would be impossible to rely on the case-control ratios to determine the disease model.

**Table 2:** Analytic framework for Section 2.1. Two SNP-pairs (where each *w*_*i*_, *v*_*j*_ denotes the probability of the respective genotype combination) and four penetrance levels (*p*_*kℓ*_, *k*, *ℓ* ∈ {0, 1}). Certain relationships among the four penetrance parameters, e.g., Eq (4), can make it impossible for us to determine an appropriate disease model for the underlying pair based on the case-control ratios.

Pair 1				Pair 2				Penetrance		
	BB	Bb	bb		DD	Dd	dd	Pair_1_\Pair_2_	R	N
AA	*w*_1_	*w*_2_	*w*_3_	CC	*v*_1_	*v*_2_	*v*_3_	R	*p*_11_	*p*_10_
Aa	*w*_4_	*w*_5_	*w*_6_	Cc	*v*_4_	*v*_5_	*v*_6_	N	*p*_01_	*p*_00_
aa	*w*_7_	*w*_8_	*w*_9_	cc	*v*_7_	*v*_8_	*v*_9_			

Since both MDR and RS rely on the case-control ratios to determine disease models, we can expect their powers (of detecting the relevant pair) to be greatly affected if Eq (4) holds, *even if only approximately*.

To offer a more concrete illustration, we simulated two examples (see Table 3). In the first one, the true disease models were the same for the two relevant SNP-pairs; in the second, they were different. The penetrance parameters *p*_10_, *p*_01_ and *p*_00_ were predetermined, and we explored a few different values for the last penetrance parameter, *p*_11_, around the value implied by Eq (4). Keep in mind, however, that unless *p*_11_ is equal to the value implied by Eq (4) *exactly*, there is still some weak signal left that is, in principle, detectable by considering the case-control ratios. The simulation was repeated for 100 times, with a total of 100 SNPs and a sample size of *n* = 800.

**Table 3:** Simulated examples for Section 2.1. Disease models for the two pairs of SNPs that contribute to the simulated outcome. The penetrance parameters, (*p*_00_, *p*_01_, *p*_10_, *p*_11_), are chosen so that the case-control ratio is the same for all genotype combinations *i* = 1, 2, …, 9 in the first pair, {A/a, B/b}.

Ex. 1: Two SNP-pairs, identical disease models (MAF = 0.3).							
	BB	Bb	bb		DD	Dd	dd
AA	0	0	1	CC	0	0	1
Aa	0	1	0	Cc	0	1	0
aa	1	0	0	cc	1	0	0
(*p*_10_ = 0.1, *p*_01_ = 0.28, *p*_00_ = 0.01 $\overset{\text{Eq}\;(4)}{\Rightarrow }$ *p*_11_ = 0.03.)							
Ex. 2: Two SNP-pairs, different disease models (MAF = 0.2).							
	BB	Bb	bb		DD	Dd	dd
AA	0	0	0	CC	0	1	1
Aa	0	1	1	Cc	1	0	0
aa	0	1	1	cc	1	0	0
(*p*_10_ = 0.09, *p*_01_ = 0.12, *p*_00_ = 0.001 $\overset{\text{Eq}\;(4)}{\Rightarrow }$ *p*_11_ = 0.016.)							

**Fig 1:**
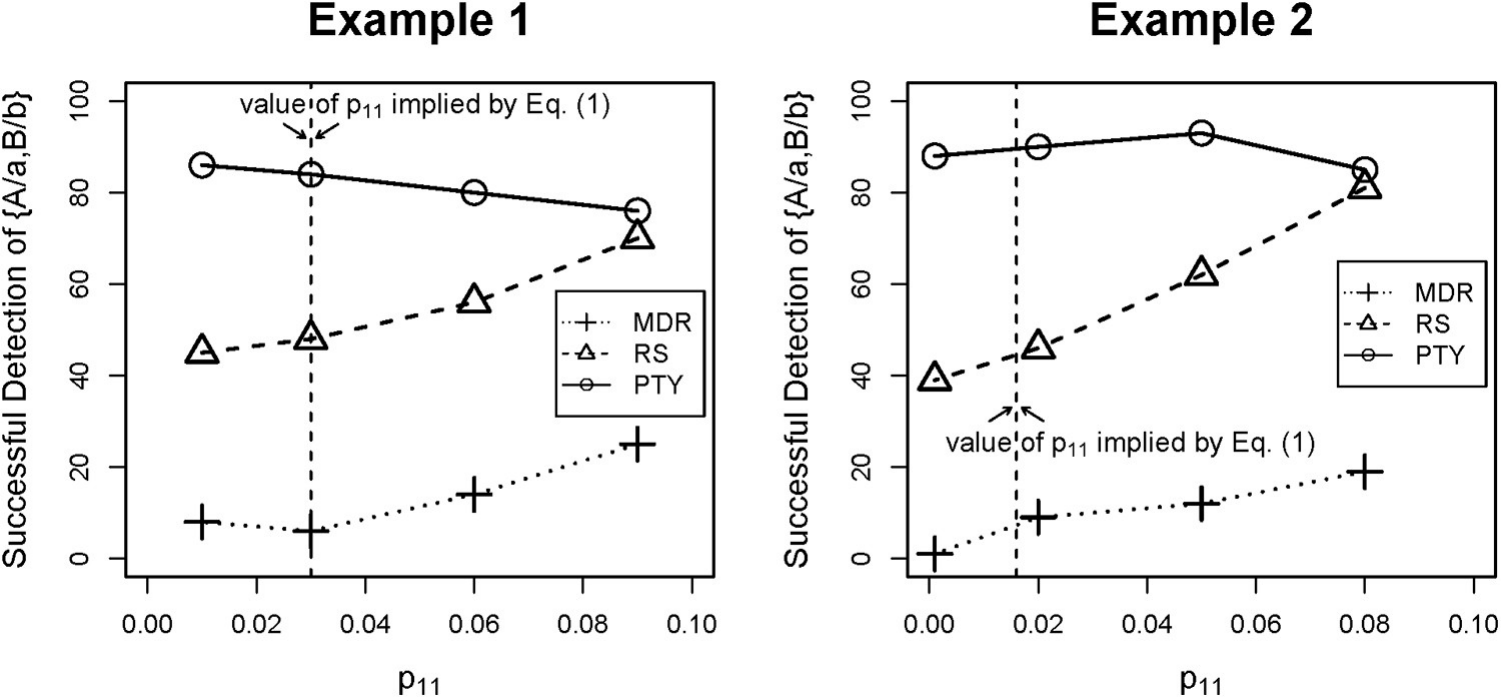
Simulated examples for Section 2.1. Number of times the first pair, {A/a, B/b}, was successfully detected (out of 100 repetitions) as the parameter *p*_11_ varied.

We then assessed the number of times each relevant pair was successfully detected by each method with the sequential screening procedure, out of 100 repetitions. A relevant pair was considered to have been successfully detected if it was among the top two pairs selected by the method. Here, the effect of the second pair ({C/c, D/d}) was stronger than the first ({A/a, B/b})—e.g., *p*_01_ > *p*_10_—and all three methods detected it perfectly (i.e., 100 times out of 100 replications), but as the parameter *p*_11_ dropped, both MDR and RS started to deteriorate in terms of their ability to detect the first pair ({A/a, B/b}), whereas our method, PTY, remained largely unaffected (Fig 1).

To better understand Eq (4), notice that it can be rearranged slightly as

$$\begin{array}{c} p_{11}=p_{01}-\frac{\sum_{j\in R}v_{j}}{\sum_{j\in N}v_{j}}\left(p_{10}-p_{00}\right). \end{array}$$ (5)

Since we typically expect *p*_10_ > *p*_00_, i.e., having a risky combination in the first pair will increase the probability of having the disease, Eq (5) implies that *p*_11_ < *p*_01_, or that having risky combinations from *both* pairs will actually lead to a *lower* probability of having the disease than having risky combinations only from the second pair. This is analogous to the logical operator, “exclusive or” (XOR).

While one certainly can argue that this may be a totally hypothetical scenario that is not likely to occur in nature, it is nonetheless a theoretical possibility against which our method, PTY, is robust.

Of course, the aforementioned XOR-type relationship means the two pairs, {A/a, B/b} and {C/c, D/d}, are interacting with each other, so there is actually a four-way interaction across the four SNPs involved. Such a high-order interaction still could be detectable by methods such as the MDR or the RS if four-way disease models were considered and screened but, as we stated earlier (Section 1), in this study we are taking a “narrow” point of view by restricting ourselves to consider only two-way interactions. Indeed, there is nothing “pathological” about having a high-order interaction; it is only “pathological” when one is restricted to consider only two-way interactions.

### 2.2 Detection of spurious effects

We have also observed that being overly adaptive to data can cause a method to be more easily tricked into detecting spurious epistatic effects, e.g., by SNPs with large individual effects. To demonstrate this, we simulated 100 SNPs on a case-control sample of size *n* = 200. Two pairs of SNPs—say, {A/a, B/b} and {C/c, D/d}—contributed to the simulated outcome independently, each according to an additive disease model (see Table 4). The SNPs A/a and C/c were simulated to have higher minor allele frequencies (MAFs) than B/c and D/d so that, according to the underlying additive disease model, they had larger marginal, individual effects than the other two.

**Table 4:** Simulated examples for Section 2.2. Disease models for the two pairs of SNPs that contribute to the simulated outcome. Numeric values (e.g., 0.1, 0.2) are penetrance parameters for the corresponding genotype combinations. (MAF = 0.5 and 0.3, respectively for the two SNPs in each pair).

	BB	Bb	bb		DD	Dd	dd
AA	0	0	0.1	CC	0	0	0.1
Aa	0	0	0.1	Cc	0	0	0.1
aa	0.1	0.1	0.2	cc	0.1	0.1	0.2

We repeated the simulation for 100 times and counted those pairs most frequently ranked by each method—with the sequential screening procedure—to be among the top two (Table 5). Both MDR and RS were more likely to select a spurious pair, {A/a, C/c}, due to the large marginal effects of both of these SNPs. They were much less effective than our method, PTY, in identifying the truly relevant pairs.

**Table 5:** Simulated examples for Section 2.2. Number of times different pairs of SNPs were among the top two pairs detected, out of 100 replications. The truly relevant pairs are emboldened.

MDR		RS		PTY	
{A/a, C/c}	75	{A/a, C/c}	74	**{A/a, B/b}**	43
{B/b, D/d}	32	{B/b, D/d}	51	**{C/c, D/d}**	42
**{C/c, D/d}**	13	{A/a, D/d}	12	{A/a, C/c}	32
{A/a, D/d}	13	{B/b, C/c}	9	{A/a, D/d}	23
**{A/a, B/b}**	10	**{C/c, D/d}**	8	{B/b, C/c}	9
{B/b, C/c}	10	**{A/a, B/b}**	7	{B/b, D/d}	6
Other Pairs	≤ 9	Other Pairs	≤ 6	Other Pairs	≤ 5

### 2.3 Exaggeration of effects and false positives

Earlier in Section 1, we already stated that both MDR and RS tend to produce many false positives. To demonstrate this point more concretely, we conducted another experiment. We simulated 100 SNPs on a case-control sample of size *n* = 400, except that, this time, *none* of the SNPs was related to the simulated outcome. We then allowed all three methods, MDR, RS, and PTY, to assess the resulting (1002)=4,950 pairs of SNPs and examined the distributional properties of the resulting association measures (see Section 1.1) produced by each method for all pairs, $\left\{{\hat{\chi }}_{i,j}^{2}:1\leq i,j\leq 100\right\}$.

**Fig 2:**
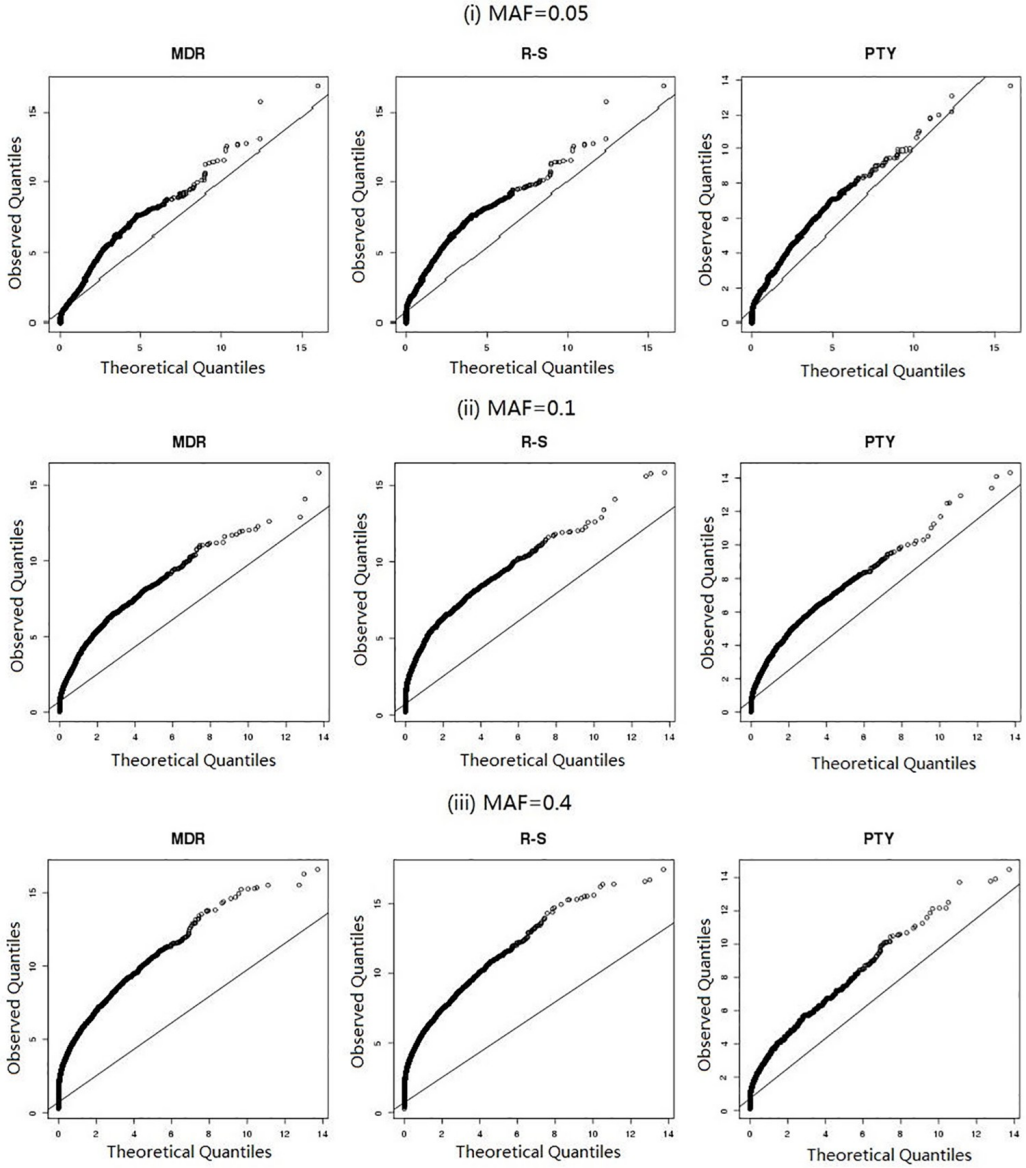
Results from simulation study (Section 2.3). Q-Q plots of nominal association measures $\left\{{\hat{\chi }}_{i,j}^{2}:1\leq i,j\leq 100\right\}$ against their theoretical quantiles.

Fig 2 shows various Q-Q plots of these association measures, produced by different methods under different MAF settings, against the theoretical quantiles of the $\chi_{\left(1\right)}^{2}$-distribution. We can see that all methods produced *inflated* association measures, leading to false discoveries. This is not a big surprise; after all, *M*_*i*,*j*_ was not just any disease model but the one deemed “best-fitting” for the underlying pair (*i*, *j*). Though the meaning of “best-fitting” differed for the three methods, a post-hoc test of independence based on *M*_*i*,*j*_ was clearly biased toward being significant. This is why we used the adjective “nominal” earlier in Section 1.1 to describe these association measures.

However, the main point here is that our method, PTY, suffered the *least* from this tendency to produce false positives. As the MAF increased, the tendency to produce false positives also became more pronounced for both MDR and RS, but not for PTY. To further make this point, we repeated the aforementioned “null simulation” 400 times. For each repetition, we computed the mean value of the (nominal) association measure,

$$\begin{array}{c} \frac{1}{4950}\sum_{i,j}{\hat{\chi }}_{i,j}^{2}, \end{array}$$ (6)

across all 4,950 SNP-pairs. The average of these mean values and its standard error over the 400 repetitions are shown in Table 6 for each method under different MAF settings. Clearly, this value is more inflated for MDR and especially for RS than it is for PTY.

**Table 6:** Results from simulation study (Section 2.3). Average values of the nominal association measures $\left\{{\hat{\chi }}_{i,j}^{2}:1\leq i,j\leq 100\right\}$ across all 4,950 SNP-pairs, together with their standard errors, over 400 repetitions.

MAF	MDR	RS	PTY
0.05	1.837 (0.229)	2.115 (0.233)	1.792 (0.214)
0.10	2.457 (0.239)	2.975 (0.230)	2.202 (0.200)
0.40	4.748 (0.289)	5.101 (0.303)	3.033 (0.221)

## 3 Method

We now describe our approach in more detail. First, we derive a metric to measure the similarity (or equivalently, difference) between two disease models. Then, we cluster all disease models into a few groups and select a prototype model from each group. Finally, we screen each pair of SNPs against the set of prototype models. The set of prototype models is decided *a priori*, without considering the disease status of individuals in the data set. This is what makes our approach less greedy, and less data-adaptive, than existing methods such as MDR and RS.

### 3.1 Similarity measure

Earlier in Section 1, we already alluded to the intuition that some disease models appear to be more similar than others (Table 1). Such intuition can be formalized in many different ways; for instance, some researchers have used a geometric approach to categorize them [17]. In this paper, we take a more pragmatic approach.

We measure the similarity of two disease models—say, *M* and *M*′—according to how much they agree in terms of their assignment of individuals into high- and low-risk groups. For *k*, *ℓ* = {0, 1}, suppose *n*_*kℓ*_ is the number of individuals classified to be high-risk by both models (*k* = *ℓ* = 1), by *M* only (*k* = 0, *ℓ* = 1), by *M*′ only (*k* = 1, *ℓ* = 0), or by neither model (*k* = *ℓ* = 0), out of a *hypothetical* group of *n*‥ individuals (Table 7). We then use the so-called Φ-coefficient [22], defined as

$$\begin{array}{c} \Phi =\frac{\left(n_{11}\right)\left(n_{00}\right)-\left(n_{10}\right)\left(n_{01}\right)}{\sqrt{\left(n_{1\cdot }\right)\left(n_{0\cdot }\right)\left(n_{\cdot 1}\right)\left(n_{\cdot 0}\right)}} \end{array}$$ (7)

to measure the concordance between *M* and *M*′. A high (low) value of Φ means the two models classify many (few) individuals to be in the same high- or low-risk group.

**Table 7:** Assignment of individuals into high- and low-risk groups by two disease models, *M* and *M*′.

*M*′\*M*	High Risk	Low Risk	Total
High Risk	*n*_11_	*n*_10_	*n*_1⋅_
Low Risk	*n*_01_	*n*_00_	*n*_0⋅_
Total	*n*_⋅1_	*n*_⋅0_	*n*_⋅⋅_

For *i* = 1, 2, …, 9, let *G*_*i*_ denote a genotype combination formed by a pair of SNPs; and let $P(D|G_{i})$ denote the penetrance (or probability of trait/disease) of the particular combination *G*_*i*_. Suppose that *M* is the true disease model with only two unique penetrance levels,

$$\begin{array}{c} P\left(D|G_{i}\right)=\{\begin{array}{cc} P_{1}, & M(G_{i})=1,\\ P_{0}, & M(G_{i})=0; \end{array} \end{array}$$ (8)

whereas *M*′ is a different disease model used in place of the true model *M*. Then, derivations contained in S2 Appendix show that the Φ-coefficient between *M* and *M*′ can be expressed as

$$\begin{array}{c} \Phi \left(M^{\prime },M\right)=\frac{\left(W_{11}\right)\left(W_{00}\right)-\left(W_{10}\right)\left(W_{01}\right)}{\sqrt{\left(\frac{U}{V}W_{11}+W_{01})(W_{10}+\frac{V}{U}W_{00})(W_{1\cdot })(W_{0\cdot }\right)}}, \end{array}$$ (9)

where

$$W_{kl}=\underset{\begin{array}{l} M\left(G_{i}\right)=k\\ M^{\prime }\left(G_{i}\right)=l \end{array}}{\sum }\mathbb{P}\left(G_{i}\right)\;\text{for}\;k,l\in \left\{0,1\right\};$$ (10)

$$U=rP_{1}\left[1-P\left(D\right)\right]+\left(1-P_{1}\right)P\left(D\right);$$ (11)

$$V=rP_{0}\left[1-P\left(D\right)\right]+\left(1-P_{0}\right)P\left(D\right);$$ (12)

*r* is the case-control ratio of the sample; and P(D) is the prevalence of the trait/disease.

Eq (9) allows us to use

$$\begin{array}{c} d\left(M^{\prime },M\right)=1-\Phi \left(M^{\prime },M\right) \end{array}$$ (13)

as a distance metric for two disease models. It is important to note here that our distance metric *d*(*M*′, *M*) is not symmetric but directional. In particular, *M* is assumed to be the true model, and *M*′ is the prototype used in its place. We shall say more about the similarity measure, Φ(*M*′, *M*), later in Section 6; here, we first give some details about how values of various parameters can be obtained in order to compute the expression on the right-hand side of Eq (9).

*W*_*kℓ*_: Assuming Hardy-Weinberg equilibrium, the MAFs of the two SNPs can be estimated from the control sample, and used to determine $P(G_{i})$ for each genotype combination *G*_*i*_ and hence *W*_*kℓ*_ as well for *k*, *ℓ* ∈ {0, 1}.

*r*: For any given data set, the case-control ratio *r* is known, e.g., *r* = 1 for a balanced case-control data set.

P(D): The prevalence, P(D), of a particular trait/disease can often be obtained from external sources, e.g., published studies and/or expert opinions. (More on this below in Sections 3.2 and 6).

*P*_1_, *P*_0_: To determine the value of these parameters, we make a convenient assumption that the underlying pair of SNPs is the actual pair associated with the outcome. Then, the prevalence is simply

$$\begin{array}{c} P\left(D\right)=\sum_{i=1}^{9}P\left(D|G_{i}\right)P\left(G_{i}\right) \end{array}$$ (14)

and the heritability (the amount of genetic contribution to overall phenotype variation [23]) is given by

$$\begin{array}{c} h^{2}=\frac{1}{\left[P(D)][1-P(D)\right]}\sum_{i=1}^{9}{\left[P\left(D|G_{i}\right)-P\left(D\right)\right]}^{2}P\left(G_{i}\right). \end{array}$$ (15)

Since we have assumed in Eq (8) that *M* has only two unique penetrance levels, i.e., each $P(D|G_{i})$ is either *P*_1_ and *P*_0_, they can now be uniquely determined from the two Eqs (14) and (15), provided that information is available about the heritability parameter, *h*^2^. This can often be obtained from external sources as well, much like the prevalence parameter. (More on this below in Sections 3.2 and 6).

### 3.2 Clustering

There are altogether 2^9^ − 2 = 510 non-trivial TTTC disease models—the trivial ones are those such that *M*(*G*_*i*_) = 1 or *M*(*G*_*i*_) = 0 for all *G*_*i*_. For clustering purposes, we need not consider disease models that are symmetric with respect to (i) the exchange of locus, i.e., swapping the two SNPs, or (ii) the exchange of disease status, i.e., flipping the binary values of each *M*(*G*_*i*_) from a zero to a one, and vice versa. The set of models that remain, which we denote as M, is listed in S3 Appendix.

**Fig 3:**
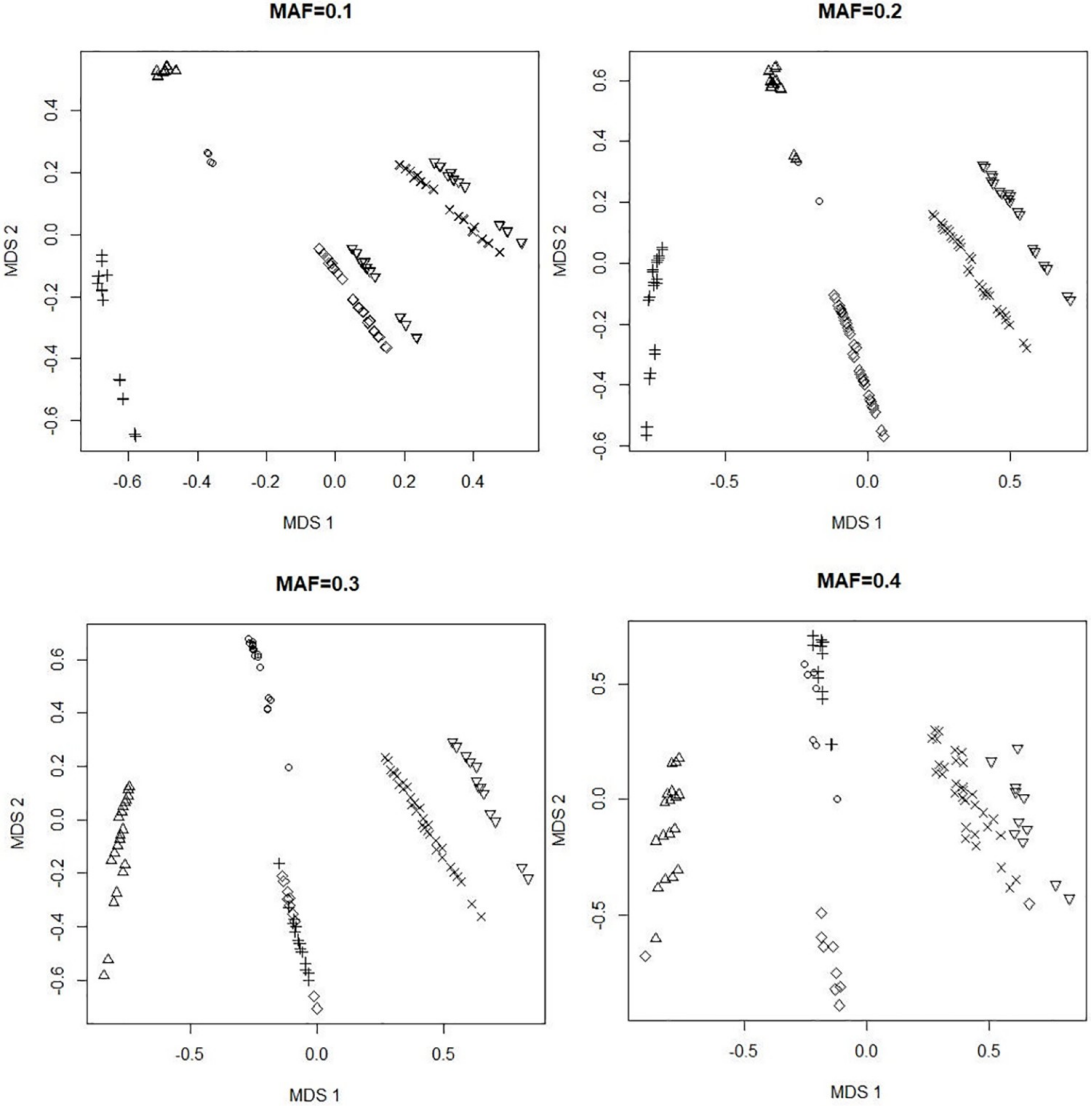
A two-dimensional map of disease models in M. The coordinates are estimated by applying the multi-dimensional scaling (MDS) technique to the symmetrized pairwise distances, *d*_*s*_(*M*_*i*_, *M*_*j*_) ≡ [*d*(*M*_*i*_, *M*_*j*_) + *d*(*M*_*j*_, *M*_*i*_)]/2, for all *i* ≠ *j*. Models clustered into the same group are depicted by the same symbol (e.g., ‘+’, ‘∘’, ‘×’). These two-dimensional coordinates explain about 50-70% of the variation in *d*_*s*_(⋅, ⋅), so there is some loss of information—in particular, some disease models may be closer to (or farther apart from) each other than how they appear in this map.

Fig 3 shows the 2-dimensional coordinates of all models ∈M as estimated by the multidimensional scaling (MDS) technique from their pairwise distances, assuming that the MAFs of both SNPs are equal to 0.1, 0.2, 0.3, and 0.4, respectively, while fixing the prevalence and heritability parameters at $P\left(D\right)=h^{2}=0.02$. While we used the directional distance metric for prototype identification (see Table 8 below), we used a symmetrized distance metric, *d*_*s*_(*M*_*i*_, *M*_*j*_) ≡ [*d*(*M*_*i*_, *M*_*j*_) + *d*(*M*_*j*_, *M*_*i*_)]/2, for performing MDS so that the resulting 2-dimensional coordinate-map (Fig 3) is more meaningful. It is clear from Fig 3 that these disease models form several clusters.

**Table 8:** The global *K*-means algorithm for identifying prototype disease models.

Let M be the set of all disease models and $M^{*}$, the prototype set (initially empty). Evaluate each $M_{i}\in M\backslash M^{*}$ as a potential new prototype, as follows: For each $M_{k}\in M\backslash \left\{M^{*}\cup M_{i}\right\}$, calculate the distances *d*(*M*_*i*_, *M*_*k*_), and $d(M_{j}^{*},M_{k})$ for all $M_{j}^{*}\in M^{*}$ if $M^{*}$ is not empty. Assign *M*_*k*_ eitder to an existing cluster—e.g., $C_{j}^{*}$, with center $M_{j}^{*}$— or to a potentially new cluster—say *C*_*i*_, with center *M*_*i*_—depending on which of *d*(*M*_*i*_, *M*_*k*_) and $d(M_{j}^{*},M_{k})$ is the shortest. After all $M_{k}\in M\backslash \left\{M^{*}\cup M_{i}\right\}$ are assigned, calculate the total within-cluster distances, $$\begin{array}{c} D\left(M_{i}\right)\equiv \sum_{M_{k}\in C_{i}}d\left(M_{i},M_{k}\right)+\sum_{M_{j}^{*}\in M^{*}}\sum_{M_{k}\in C_{j}^{*}}d\left(M_{j}^{*},M_{k}\right), \end{array}$$ as a result of using *M*_*i*_ as an additional cluster center. Identify a new prototype model as the one that minimizes the total within-cluster distances, i.e., $$\begin{array}{ccc} M^{*} & = & \underset{M_{i}\in M\backslash M^{*}}{\text{arg}\;\text{min}}\;D\left(M_{i}\right), \end{array}$$ and insert it into the set $M^{*}\leftarrow M^{*}\cup M^{*}$. Repeat steps 2-3 until a certain number of prototypes are identified.

One can easily expect from Eq (9) that our distance metric will be affected by the MAFs of the underlying SNPs, but Fig 3 shows that the resulting clusters do not change significantly. Therefore, it is not necessary to repeat the prototype selection step for every individual SNP-pair. Instead, we simply discretized the MAF-scale into 6 bins, {0.05, 0.1, 0.2, 0.3, 0.4, 0.45}, and created 36 different *sets* of prototypes for all 6 × 6 pairwise combinations. For example, when screening a SNP-pair (*i*, *j*) with (MAF_*i*_, MAF_*j*_) = (0.068, 0.182), we would use the set of prototypes for (MAF_*i*_, MAF_*j*_) = (0.05, 0.2), and so on.

We also examined similar plots (not shown) produced with different values of P(D) and *h*^2^. While these parameters also affected the distance metric, they did not produce any substantial changes to the clustering. Intuitively, this is because there has to be a fairly drastic warping of the relative distances between objects in order to alter their grouping; we shall come back to this point again later in Section 6. Hence, for this paper we simply used $P\left(D\right)=h^{2}=0.02$.

**Fig 4:**
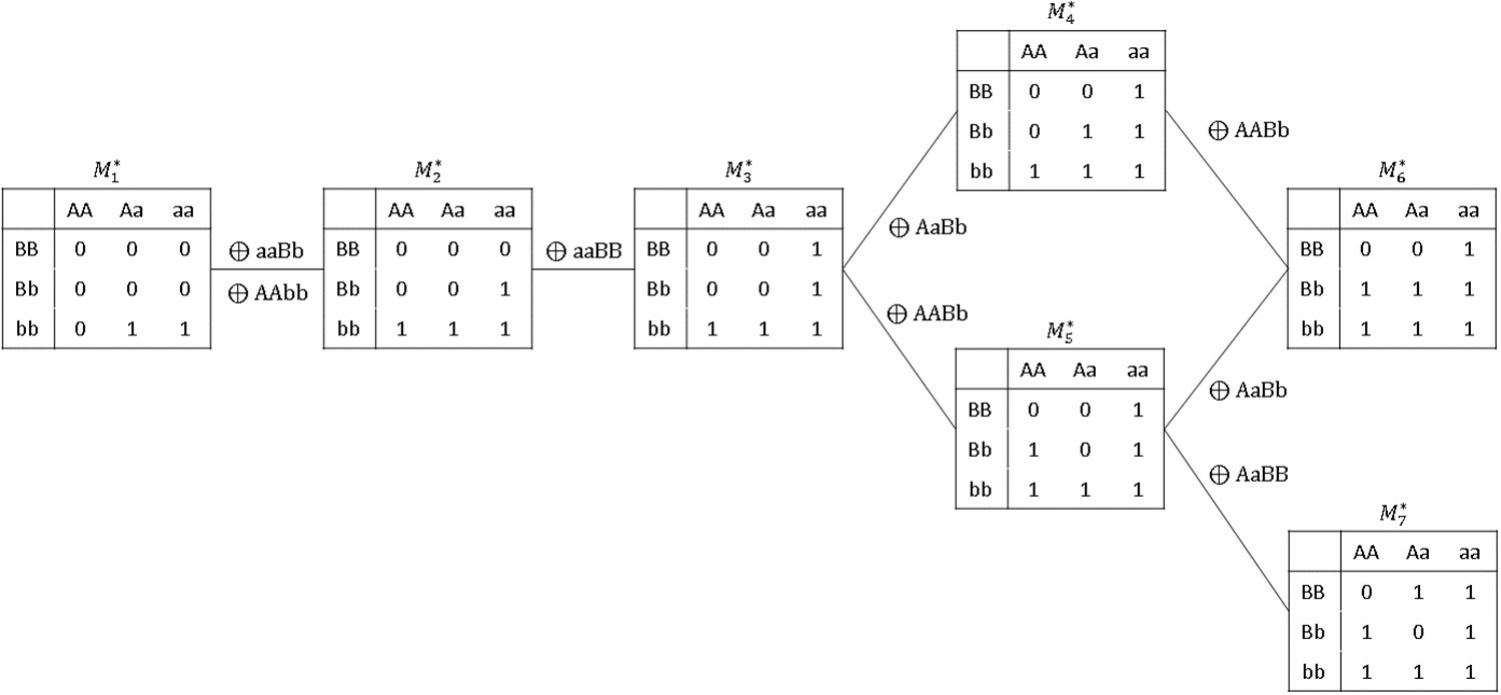
The set of prototype disease models selected by the global *K*-means algorithm (*K* = 7) for SNP-pairs (*i*, *j*) with (MAF_*i*_, MAF_*j*_) ≈ (0.2, 0.2). The structural relationships between the seven prototypes were manually annotated; the clustering algorithm itself was not capable of making this type of discoveries.

In principle, we could use any distance-based clustering algorithm. In our implementation, we used the “global *K*-means” algorithm [24]. The steps of our algorithm are given in Table 8. Based on Fig 3, we selected 7 prototypes for each MAF-combination. As an illustration, the prototypes for SNP-pairs with (MAF_*i*_, MAF_*j*_) = (0.2, 0.2) are displayed in Fig 4 with manual annotations to reveal their relationships with one another. One may interpret this figure to mean that, for a pair of SNPs, both of which have MAF around 0.2, these are the primary epistatic effects to consider, and their structural relationships; any other will likely be very similar to one of these—in terms of how they would classify individuals into high- versus low-risk groups, that is. This is also a unique piece of insight from our overall methodology that is not otherwise available from MDR or RS.

Finally, it is worth emphasizing that the reduction of disease models to the set M, due to various symmetry considerations we mentioned in the opening paragraph of this subsection, is *only* applicable to the clustering and prototype selection stage. When screening each candidate SNP-pair, prototype disease models that are asymmetric with respect to the exchange of locus, such as $M_{1}^{*}$ in Fig 4, are always tested both for {A/a,B/b} and for {B/b,A/a}, and so on.

## 4 Simulation study

To motivate our approach, we already presented a few simulated examples in Section 2, where we concentrated on evidence that our approach appears to overcome various weaknesses of existing approaches. In this section, we assess our approach more generally with a number of simulated examples that are commonly examined in the literature.

### 4.1 Set-up

In each simulation, we generated 100 SNPs, but only the first two determined the simulated outcome according to a particular disease model (more details below in Section 4.2). To evaluate the performance of a method, we used a metric known as the F-measure, defined as

$$\begin{array}{c} \text{F-measure}\equiv \frac{2\times (\text{precision})\times (\text{recall})}{\left(\text{precision})+(\text{recall}\right)}, \end{array}$$ (16)

where

$$\begin{array}{c} \text{precision}=\{\begin{array}{cc} \frac{1}{\#(\text{pairs}\;\text{detected})}, & \text{if}\;\text{the}\;\text{true}\;\text{pair}\;\text{was}\;\text{detected},\\ 0, & \text{otherwise}; \end{array} \end{array}$$ (17)

and

$$\begin{array}{c} \text{recall}=\{\begin{array}{cc} 1, & \text{if}\;\text{the}\;\text{true}\;\text{pair}\;\text{was}\;\text{detected},\\ 0, & \text{otherwise}. \end{array} \end{array}$$ (18)

Each simulation was repeated for 400 times, and the average F-measure and its standard error were recorded (Table 11). To avoid excessive computation, we used the marginal screening procedure for all methods; see Section 1.1.

The F-measure is a widely used criterion in the field of information retrieval; it is a single numeric metric that balances the trade-off between true positives and false positives. We adopted the F-measure, instead of other metrics such as the “balanced accuracy”, because the underlying problem really is more of an “information retrieval” problem than a “classification” problem, not only because there are far more true negatives than true positives, but also because detecting the positives—here, the relevant SNP-pair—is a much more important objective than correctly calling out the negatives. Imagine the experience of conducting a Google search. For each given query, most of the web pages on the Internet are irrelevant. Therefore, from a customer’s perspective, the most important measure must concentrate on the set of detected web pages retrieved by the search engine, for example, the top twenty. How many of these are relevant (true positives), and how many are irrelevant (false positives)? By and large, the customer does *not* care how many truly irrelevant web pages have been correctly left out of the search result—that is, the customer does not care about the true negatives. Moreover, because the set of truly irrelevant pages is so large, the true negative rate will also be difficult to distinguish meaningfully for most “reasonable” search engines; any “reasonable” search engine will have a true negative rate of >99%. Therefore, measures like the “balanced accuracy” actually places an undue amount of emphasis on this rather inconsequential side of the performance. This is also why the information retrieval community tend to largely favor metrics such as the F-measure to those more commonly used by the classification community, such as “balanced accuracy”. The situation of detecting relevant SNP-pairs is very much akin to performing a Google search in that (i) most pairs are not signals; (ii) we care *not very much* about getting the true negatives right; (iii) instead, we care *a lot* about how many of the detected pairs are true positives or false positives.

### 4.2 Disease models

Our primary focus was to evaluate the ability of different methods to detect different epistatic effects as represented by different disease models.

First, we included six disease models with main effects (Table 9). They were among the most commonly used examples in various studies [25–30]. Here in Table 9, these models are parameterized in terms of odds, $P\left(D|G_{i}\right)/\left[1-P\left(D|G_{i}\right)\right]$, rather than penetrance, $P(D|G_{i})$. The parameters *α* and *θ* were determined by simultaneously solving Eqs (14) and (15), given the prevalence P(D) and heritability *h*^2^ of the outcome, as well as the MAF of each SNP. We simply fixed P(D)=0.02, but repeated each of these simulations with MAF = 0.1 and 0.4 for all SNPs. Assuming Hardy-Weinberg equilibrium, the MAF determined $P(G_{i})$ for each genotype combination *G*_*i*_, leaving *α* and *θ* to be the only unknowns in Eqs (14) and (15) so that they could be uniquely determined.

**Table 9:** Simulated examples for Section 4. Disease models with main effects. The parameters *α* and *θ* were uniquely determined given prevalence P(D), heritability *h*^2^, and MAF. We fixed P(D)=0.02, and repeated each simulation with MAF = 0.1 and 0.4 for all SNPs.

(a) Threhold (T) *h*^2^ = 0.02				(b) Dominant-Dominant (DD) *h*^2^ = 0.02			
	BB	Bb	bb		BB	Bb	bb
AA	*α*	*α*	*α*	AA	*α*	*α*	*α*
Aa	*α*	*α*	*α*(1 + *θ*)	Aa	*α*	*α*(1 + *θ*)	*α*(1 + *θ*)
aa	*α*	*α*(1 + *θ*)	*α*(1 + *θ*)	aa	*α*	*α*(1 + *θ*)	*α*(1 + *θ*)
(c) Modifying Effect (MOD) *h*^2^ = 0.02				(d) Exclusive Or (XOR) *h*^2^ = 0.02			
	BB	Bb	bb		BB	Bb	bb
AA	*α*	*α*	*α*	AA	*α*	*α*	*α*(1 + *θ*)
Aa	*α*	*α*	*α*(1 + *θ*)	Aa	*α*	*α*	*α*(1 + *θ*)
aa	*α*(1 + *θ*)	*α*(1 + *θ*)	*α*(1 + *θ*)	aa	*α*(1 + *θ*)	*α*(1 + *θ*)	*α*
(e) Multiplicative (ME) *h*^2^ = 0.015				(f) Threshold Multiplicative (MET) *h*^2^ = 0.015			
	BB	Bb	bb		BB	Bb	bb
AA	*α*	*α* (1 + *θ*)	*α*(1 + *θ*)^2^	AA	*α*	*α*	*α*
Aa	*α* (1 + *θ*)	*α*(1 + *θ*)^2^	*α*(1 + *θ*)^3^	Aa	*α*	*α* (1 + *θ*)	*α*(1 + *θ*)^2^
aa	*α*(1 + *θ*)^2^	*α*(1 + *θ*)^3^	*α*(1 + *θ*)^4^	aa	*α*	*α*(1 + *θ*)^2^	*α*(1 + *θ*)^4^

Next, we included four disease models without main effects (Table 10), taken from an earlier study conducted by Ritchie *et al*. [31], in which these disease models were created to have purely epistatic effects in the sense that no marginal effect existed for either SNP involved.

**Table 10:** Simulated examples for Section 4. Disease models without main effects, taken from [31], where they were specifically constructed in such a way that there is no individual association between either SNP and the disease.

(a) DMN 1 MAF = 0.25, *h*^2^ = 0.016				(b) DMN 2 MAF = 0.25, *h*^2^ = 0.04			
	BB	Bb	bb		BB	Bb	bb
AA	0.08	0.07	0.05	AA	0	0.1	0.09
Aa	0.1	0	0.1	Aa	0.04	0.01	0.08
aa	0.03	0.1	0.04	aa	0.07	0.09	0.03
(c) DMN 3 MAF = 0.1, *h*^2^ = 0.002				(d) DMN 4 MAF = 0.1, *h*^2^ = 0.015			
	BB	Bb	bb		BB	Bb	bb
AA	0.07	0.05	0.02	AA	0.09	0.001	0.02
Aa	0.05	0.09	0.01	Aa	0.08	0.07	0.005
aa	0.02	0.01	0.03	aa	0.003	0.007	0.02

The disease models, T, DD, MOD and XOR, all have two penetrance levels (Table 9), and so do our prototype disease models (see Fig 4). However, in designing our simulations we took care to ensure that, while some of these models (e.g., XOR) were relatively close to a prototype, others (e.g., DD) were relatively far from all prototypes, as measured by our metric Φ. The disease models, ME, MET, and DMN 1-4, on the other hand, all have more than two penetrance levels (Tables 9 and 10). They were chosen so that a wider variety of epistatic effects could be studied.

### 4.3 Thresholds

The nominal association measures produced by different methods for each pair of SNPs (see Section 1.1) were thresholded by their corresponding (nominal) p-values,

$$\begin{array}{c} {\hat{p}}_{i,j}\equiv P\left(\chi_{\left(1\right)}^{2}>{\hat{\chi }}_{i,j}^{2}\right), \end{array}$$ (19)

and a pair was considered “detected” if ${\hat{p}}_{i,j}<\alpha$, where *α* was a significance threshold. For convenience, we applied simple Bonferroni corrections to determine the threshold *α*. As there were a total of (1002)=4,950 pairs of SNPs, it was natural to first consider a threshold of

$$\begin{array}{c} \alpha^{\text{easy}}=0.05÷4,950\approx 10^{-5}. \end{array}$$ (20)

To account for the fact that these nominal association measures were biased (see Section 2.3), however, we also considered applying a more stringent threshold. But since there was not a clear way to pick such a threshold that easily could be considered “fair” for all methods, as each method considers a different number of (almost certainly) correlated disease models for a pair of SNPs, we simply settled on a convenient choice of

$$\begin{array}{c} \alpha^{\text{hard}}=0.05÷4,950÷8\approx 1.26\times 10^{-6}, \end{array}$$ (21)

based on the fact that RS would always consider 8 different disease models. Correcting significance thresholds for simultaneous tests of correlated hypotheses is an intricate inferential problem, for which there is no good solution yet. It is not clear whether *α*^hard^ is really the “correct” threshold for RS but, as a *rough* guideline, one may think that this choice would favor RS slightly. Our empirical results below do support this interpretation to some extent.

### 4.4 Results

Results are given in Table 11. We used a relatively large sample size of *n* = 600 when the MAF was relatively low (e.g., 0.1, 0.25), and a relatively small sample size of *n* = 300 when it was high (e.g., 0.4). This is because, when the MAF was relatively high (low), the underlying signals became stronger (weaker) and easier (harder) to detect, and all the methods would perform quite well (badly) if given a sample that was “too large (small)”, making it difficult to tell them apart. For our simulated cases with 100 SNPs, we found that all methods essentially became indistinguishable when the sample size reached as low as *n* = 100 or as high as *n* = 1000.

**Table 11:** Results from simulation study (Section 4). Average F-measures (and their standard errors) over 400 replications. A star (*) in front of the number indicates the best performer for that simulation.

			*α*^easy^ = 1.00 × 10^−5^						*α*^hard^ = 1.26 × 10^−6^					
*n*	MAF	Model	MDR		RS		PTY		MDR		RS		PTY	
600	0.1	T	0.012	(0.005)	0.080	(0.013)	*0.083	(0.015)	0.000	(0.000)	*0.046	(0.012)	0.030	(0.010)
		MOD	0.062	(0.009)	0.067	(0.007)	*0.075	(0.010)	0.063	(0.011)	*0.109	(0.013)	0.090	(0.013)
		DD	0.183	(0.016)	0.172	(0.015)	*0.278	(0.019)	0.341	(0.023)	0.372	(0.021)	*0.449	(0.024)
		XOR	0.199	(0.014)	0.198	(0.013)	*0.328	(0.020)	0.283	(0.022)	0.449	(0.022)	*0.531	(0.024)
		ME	0.011	(0.000)	0.011	(0.000)	*0.012	(0.000)	0.013	(0.000)	0.013	(0.001)	*0.015	(0.001)
		MET	0.234	(0.019)	0.245	(0.016)	*0.294	(0.021)	0.335	(0.025)	*0.414	(0.023)	0.350	(0.024)
300	0.4	T	0.167	(0.011)	0.152	(0.010)	*0.223	(0.014)	*0.357	(0.017)	0.346	(0.017)	0.317	(0.018)
		MOD	0.076	(0.007)	0.064	(0.006)	*0.110	(0.010)	0.163	(0.013)	0.154	(0.013)	*0.202	(0.015)
		DD	0.015	(0.001)	0.014	(0.001)	*0.135	(0.009)	0.035	(0.005)	0.032	(0.005)	*0.276	(0.014)
		XOR	0.195	(0.012)	0.164	(0.011)	*0.306	(0.016)	0.441	(0.018)	0.409	(0.018)	*0.561	(0.019)
		ME	0.022	(0.002)	0.020	(0.002)	*0.033	(0.002)	0.043	(0.004)	0.039	(0.003)	*0.086	(0.006)
		MET	0.073	(0.006)	0.074	(0.007)	*0.076	(0.008)	0.132	(0.011)	*0.141	(0.011)	0.103	(0.010)
600	0.25	DMN 1	0.729	(0.015)	0.686	(0.016)	*0.935	(0.009)	0.951	(0.008)	0.939	(0.009)	*0.992	(0.003)
	0.25	DMN 2	0.743	(0.015)	0.705	(0.016)	*0.938	(0.010)	0.959	(0.007)	0.944	(0.008)	*0.972	(0.009)
	0.1	DMN 3	0.700	(0.018)	0.675	(0.018)	*0.832	(0.019)	0.822	(0.021)	*0.852	(0.019)	0.722	(0.026)
	0.1	DMN 4	0.752	(0.015)	0.720	(0.015)	*0.897	(0.014)	0.912	(0.013)	*0.921	(0.012)	0.831	(0.021)

As we explained previously, the threshold, *α*^easy^, only includes a simple Bonferroni correction—here, for multiple testing of 4,950 pairs—and does not account for the fact that a method, whether MDR, RS, or PTY, has usually tested a few disease models already before testing the significance of the SNP-pair against the outcome. Strictly speaking, therefore, the Bonferroni correction alone is not enough, and often leads to inflated false positive rates. Among the three methods, PTY is the least prone to false positives, which explains why its performance is the best under *α*^easy^. Generally speaking, our results confirm that there is some practical value to consider a less greedy and less data-adaptive procedure such as ours for epistasis detection.

### 4.5 Comments

Throughout our simulation study, we have assessed the performance of each screening method by its ability to detect the underlying SNP-pair, but not by whether the true disease model is correctly identified as well. The detection of the relevant SNP-pair is certainly the more fundamental task. Once the relevant SNP-pairs are identified, further studies can be conducted to determine the real underlying mechanism. Such an approach is certainly not unusual in the context of genome-wide association (GWA) studies. For most GWA studies in the literature, single SNPs are often tested and reported using disease models—e.g., additive, dominant, and so on—that are not necessarily the correct ones. Ascertaining the true disease model is almost never the goal of the initial GWA study; detecting the affected SNPs is.

In fact, this is also the very reason why our method works, because one need *not* always use exactly the true disease model in order to detect a pair of affected SNPs. While using a “very wrong” disease model can negatively affect the chances of detecting an affected SNP-pair, one has a good chance of making the detection as long as the disease models used for screening is “close enough” to the true one. Due to the way our prototype models are selected—i.e., as representative models from each cluster, there is a very good chance that at least one of our models is “close enough” to the true one.

## 5 Analysis of bipolar disorder data

In this section, we report our analysis of the phase I bipolar disorder data from the Wellcome Trust Case Control Consortium (WTCCC) [32]. Because our method is aimed at screening SNP-pairs for different epistatic effects (rather than individual SNPs for main effects), we focus on the *complementary value* that our method offers—in particular, its ability to find relevant SNPs that other methods may still miss.

The WTCCC project involves genotyping of 500K SNPs on humans of British ancestry. Bipolar disorder is one of seven diseases being studied by the WTCCC, and the shared control samples consist of 1, 500 individuals from the 1958 British Birth Cohort and another 1, 500 individuals selected from blood donors recruited as part of their project.

Identical-twin studies have shown that bipolar disorder has a strong genetic component [33]. Current findings from genome-wise association studies (GWAS) demonstrate that bipolar disorder shares many genetic overlaps with schizophrenia and other major depressive disorders, and that it is also characteristic of being polygenic, i.e., many variants that coalesce into functional pathways contribute to the disorder with small effects. The current understanding of its neurobiology is that changes in inflammatory cytokines, corticosteroids, neurotrophins, mitochondrial energy generation, oxidative stress, and neurogenesis are all involved in a comprehensive way to explain its various clinical features [34].

### 5.1 Pre-processing

We began by applying the same data quality control procedures as described in [32]—excluding SNPs with > 5% missing observations (> 1% for SNPs with MAF < 0.05), Hardy-Weinberg exact p-value < 5.7 × 10^−7^, p-value < 5.7 × 10^−7^ for either a one- or two degree-of-freedom test of association between the two control groups, and genome-wide heterozygosity < 23% or > 30%; as well as samples with > 3% missing across all SNPs. In addition, we also filtered out SNPs with MAF < 1%, p-value < 10^−7^ for a univariate test of association, and p-value < 10^−5^ for a test of Hardy-Weinberg equilibrium. The remaining data contained 1, 868 cases (individuals with bipolar disorder), 2, 938 controls, and 405, 524 SNPs. Eliminating “easily detectable” SNPs with “obvious” main effects is not uncommon for studies that focus on the detection of SNP-SNP interactions—for example, the paper by Wan *et al*. [15] that proposed the RS method also did this.

### 5.2 Mapping SNPs to genes

We used the marginal screening procedure (see Section 1.1) to screen and rank all pairs of SNPs. Here, we focus on the 100 unique SNPs appearing in the top 85 pairs (nominal p-value < 10^−11^). We used the “Ensembl gene annotation system” [35] as well as SNPnexus [36] to map these SNPs to the genes in which they most likely reside. Altogether, we identified 75 genes in this manner.

**Fig 5:**
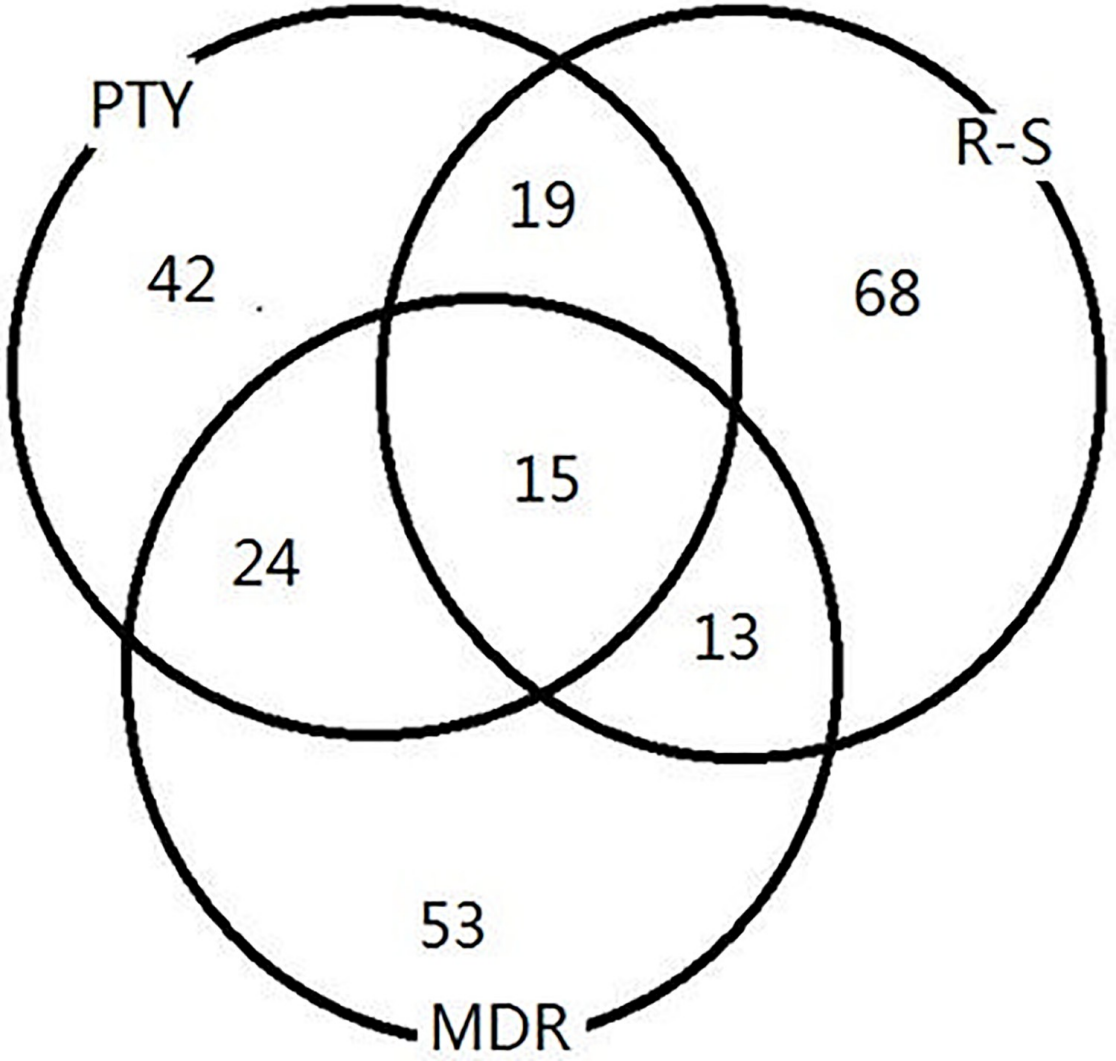
Analysis of bipolar disorder data. Venn diagram of unique SNPs appearing in the top 85 pairs detected by PTY, RS, and MDR, respectively. SNPs detected multiple times (e.g., occurring in multiple pairs) were counted only once.

Fig 5 shows the number of SNPs appearing in the top 85 pairs identified by PTY, MDR and RS, respectively. While 15 SNPs were identified by all three methods, 42 were identified by our method alone and they were mapped to 18 unique genes. Five of them—specifically, UNC13A [37], RGS6 [38], DPP10 [39], FGF14 [40] and TLE4 [40]—had been associated with bipolar disorder or related suicide attempts. Moreover, the SNP that was mapped to FGF14 had a p-value of 0.03 on a *univariate* test of association, indicating that it would have had no chance of being detected in a genome-wide screening of individual SNPs. Here, it was detected as a result of pairwise screening that focused on epistatic effects.

**Fig 6:**
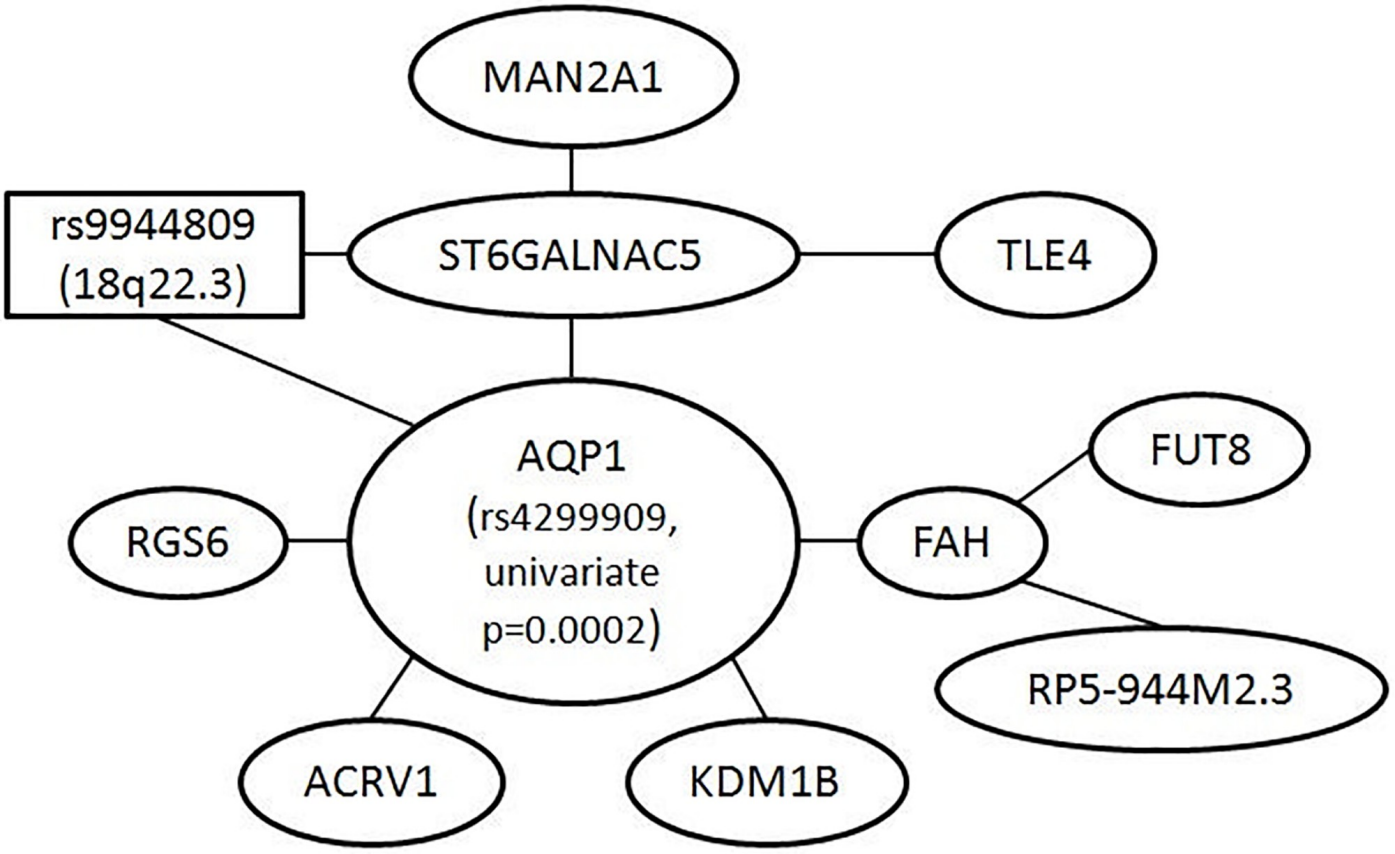
Analysis of bipolar disorder data. Largest interaction network formed by genes mapped from SNPs appearing in the top 85 pairs. Each node is either a gene (oval), or a SNP (rectangle) itself if it cannot be mapped to any gene. The size of the node is irrelevant—it is determined by the amount of text inside rather than anything scientific. A link between two nodes means the SNPs underlying the nodes are from the same pair detected, so, for example, a link between AQP1 and FAH means that a pair of SNPs—one of which was mapped to AQP1 and another, to FAH—was among the top 85 pairs detected. The resulting network contains many disjoint components. The one presented here is the biggest component.

Fig 6 shows the largest interaction network based on the 75 genes we identified. The hub gene, AQP1, encodes a small integral membrane protein that functions as a water channel protein and is potentially involved in a human neurological disorder called “central pontine myelinolysis” [41]. The specific SNP that was mapped to this gene (rs4299909) had a p-value of 0.0002 based on a univariate test of association; hence, it would have had no chance of being detected by marginal screening of individual SNPs, either. Here again, it was detected as a result of pairwise screening that focused on epistatic effects.

Among other genes in this network, ST6GALNAC5 is known to catalyse the transfer of sialic acid to cell surface proteins, and sialic acid has been suggested as an essential nutrient for brain development and cognition [42]. RGS6 regulates G protein signaling and may modulate neuronal activities; in previous studies, SNPs in this gene have been reported to be associated with schizophrenia [43]. MAN2A1 encodes a glycosyl hydrolase (a common enzyme) and catalyses the final hydrolytic step in the N-glycan maturation pathway; many SNPs in this gene have been reported to be associated with various phenotypes and diseases, including Alzheimer’s disease [44, 45]. TLE4 inhibits the transcriptional activation mediated by PAX5, and by CTNNB1 and TCF family members in Wnt signaling, which has been suggested to be potentially involved in the pathophysiology of bipolar disorder [46]. FAH encodes the last enzyme in the tyrosine metabolism pathway; the amino acid, tyrosine, is a precursor to neurotransmitters and increases plasma neurotransmitter levels—particularly dopamine and norepinephrine, both important neurotransmitters in the brain [47]. FUT8 encodes an enzyme belonging to the family of fucosyltransferases; a variant in this gene has been reported to influence glutamate concentrations in brains of patients with multiple sclerosis [48]—glutamate is a neurotransmitter accounting in total for well over 90% of the synaptic connections in the human brain.

Out of the 75 genes we identified, the following have also been reported by various independent studies to be associated with bipolar disorder, or suicides related to bipolar disorder: ANK3 [49], CNTNAP2 [50], PTPRN2 [51], DSCAM [37], PSD3 [37], RAPGEF4 [52], CPN1 [53], EPHB2 [40], CAP2 [40], NAV2 [40], and ABCB1 [40].

### 5.3 Gene set enrichment analysis

To further validate our findings, we also performed gene set enrichment analysis (GSEA) [54] on the aforementioned set of 75 genes. GSEA identifies classes of genes (e.g., those involved in specific pathways) that are over-represented in a given gene set (e.g., the ones we discovered) and may have an association with disease phenotypes, by comparing the candidate set against background databases. Gene Ontology [55] is one such database, which annotates and classifies genes in terms of their associated biological processes, cellular components and molecular functions. Other popular databases include KEGG [56] and Pathway Commons [57]. To compare a candidate gene set to various background databases and determine whether certain gene groups (e.g., those occurring in known pathways) appear statistically more or less often than expected, we used a tool called WebGestalt [58].

Table 12 lists the statistically enriched pathways from KEGG (multiple-testing adjusted p-value ≤ 0.05). Many of them have been associated with bipolar disorder or related diseases. For instance, the neurotransmitter dopamine, which is believed to have connections to bipolar disorder, is part of the tyrosine metabolism pathway (line 3). The N-Glycan biosynthesis pathway (line 4) has been reported to be significantly enriched by a study of bipolar disorder in Canadian and UK populations [59]. Both arginine and proline (line 5) have been related to schizophrenia [60]. The ErbB signaling pathway (line 7) regulates a diverse range of physiological responses, such as cell proliferation, migration, differentiation, apoptosis and motility; and insufficient ErbB signaling has been associated with the development of neuro-degenerative diseases in humans [61]. The regulations of the lysosome pathway (line 9) and of the actin cytoskeleton pathway (line 14) were found in a transcriptome sequencing and GWA study to be statistically enriched in genes associated with schizophrenia [62].

**Table 12:** Analysis of bipolar disorder data. GSEA results from KEGG. O = number of genes in the discovered set; C = total number of genes in the given pathway.

Line	Name	O	C	p-value	
				Nominal	Adjusted
1	metabolic pathways	13	1130	≪ 0.01	≪ 0.01
2	thyroid cancer	2	29	< 0.01	0.01
3	tyrosine metabolism	2	41	< 0.01	0.01
4	N-glycan biosynthesis	2	49	< 0.01	0.01
5	arginine and proline metabolism	2	54	< 0.01	0.01
6	melanoma	2	71	0.01	0.02
7	ErbB signaling pathway	2	87	0.01	0.02
8	hepatitis C	2	134	0.02	0.03
9	lysosome	2	121	0.02	0.03
10	axon guidance	2	129	0.02	0.03
11	pathways in cancer	3	326	0.02	0.03
12	cell adhesion molecules	2	133	0.02	0.03
13	endocytosis	2	201	0.05	0.05
14	regulation of actin cytoskeleton	2	213	0.05	0.05

For comparison, the corresponding results for MDR and RS are provided in S4 Appendix, while enriched pathways from Gene Ontology and Pathway Commons (for PTY identified genes only) are provided in S5 Appendix.

## 6 Discussion

This paper is concerned with screening pairs of SNPs, rather than just individual SNPs, for their association with various phenotypes. The complication is that there are many mechanisms—corresponding to different epistatic effects and described by different disease models—for a pair of SNPs to be associated with the outcome.

At the highest level, our main point is that we would be better off using less greedy approaches to determine the “best” disease model for each pair of SNPs. While there are certainly many different ways to achieve this goal, some of which are currently under our active investigation, in this paper we have concentrated on the simple idea of first clustering the disease models and then limiting the candidates to a set of prototypes selected from each respective cluster.

Earlier in Section 1, we stated that screening for higher-order interactions at a genome-wide level is still largely impractical at the present time, but when the time does become ripe for doing so, we think our idea of using prototype disease models will become even more attractive because, as higher-order interactions are considered, there will be combinatorial growth in the number of disease models and a heightened tendency for greedy approaches to produce false positives.

Prototype disease models can be selected in many different ways, although we do not expect that using different sets of prototypes will make a substantial difference. The specific proposal we have outlined in this paper is based on using a particular metric, Φ(*M*′, *M*), to quantify the similarity of disease models. We now say more about the intuitive appeal of this metric, as promised earlier in Section 3.1.

Let $r_{0}=P\left(D\right)/\left[1-P\left(D\right)\right]$ denote the population-wide case-control ratio. Then, the ratio *U*/*V* appearing in the denominator of Eq (9) is simply

$$\begin{array}{c} \frac{U}{V}=\frac{\left(P_{1}\right)r+\left(1-P_{1}\right)r_{0}}{\left(P_{0}\right)r+\left(1-P_{0}\right)r_{0}}=\frac{r_{0}+\left(r-r_{0}\right)P_{1}}{r_{0}+\left(r-r_{0}\right)P_{0}}. \end{array}$$ (22)

This makes it clear that, if *r* = *r*_0_, then *U*/*V* = 1. In this case, it is easy to see that the denominator of the Φ-coefficient can be interpreted as $\sqrt{V\text{ar}\left(M^{\prime }\right)V\text{ar}\left(M\right)}$. This is because *M* can be viewed as a Bernoulli random variable mapping various genotype combinations to either 0 or 1, with $P\left(M=1\right)=W_{1\cdot }$ and $P\left(M=0\right)=W_{0\cdot }$, so $V\text{ar}\left(M\right)=W_{1\cdot }W_{0\cdot }$. Likewise,

$$\begin{array}{ccc} V\text{ar}(M^{\prime }) & = & W_{\cdot 1}W_{\cdot 0}\\ & = & \left(W_{11}+W_{01}\right)\left(W_{10}+W_{00}\right)\\ & = & \underset{M=1}{\underset{︸}{W_{11}W_{10}}}+\underset{M\neq M^{\prime }}{\underset{︸}{W_{01}W_{10}}}+\underset{M=M^{\prime }}{\underset{︸}{W_{11}W_{00}}}+\underset{M=0}{\underset{︸}{W_{01}W_{00}}}. \end{array}$$ (23)

We can decompose $V\text{ar}(M^{\prime })$ into four terms, as shown above in Eq (23), where each successive term can be seen to measure the variability in *M*′ when *M* = 1, when *M* and *M*′ completely disagree, when they completely agree, and when *M* = 0, respectively.

However, for a case-control sample, it is often the case that *r* ≫ *r*_0_, in which case Eq (22) implies that *U*/*V* ≈ *P*_1_/*P*_0_ > 1. We can now see that, in this case, Eq (9) implicitly tells us to calculate $V\text{ar}(M^{\prime })$, the variance of the potential prototype model *M*′ used to approximate/represent *M*, differently:

$$\begin{array}{c} V\text{ar}\left(M^{\prime }\right)=\frac{U}{V}W_{11}W_{10}+W_{01}W_{10}+W_{11}W_{00}+\frac{V}{U}W_{01}W_{00}. \end{array}$$ (24)

In particular, among genotypes considered to be risky by *M* (the set for which *M* = 1), the variability in *M*′ should be up-weighted, which reduces their similarity; whereas, among those considered to be non-risky by *M* (the set for which *M* = 0), the variability in *M*′ should be down-weighted, which increases their similarity. In other words, when considering *M*′ as a potential prototype for representing *M*, the metric Φ(*M*′, *M*) “thinks” it is more important for *M*′ to agree with *M* on their assignments of the risky genotypes than for them to agree on the non-risky ones. This is intuitively appealing; a concrete numeric example is given in S6 Appendix.

The approximation that *U*/*V* ≈ *P*_1_/*P*_0_ also allows us to see more clearly how the parameters, P(D) and *h*^2^, affect the metric Φ. The solution to Eqs (14) and (15) is:

$$\begin{array}{c} P_{1}=P\left(D\right)+\sqrt{\frac{W_{0\cdot }}{W_{1\cdot }}P\left(D\right)\left[1-P\left(D\right)\right]h^{2}},\;P_{0}=P\left(D\right)-\sqrt{\frac{W_{1\cdot }}{W_{0\cdot }}P\left(D\right)\left[1-P\left(D\right)\right]h^{2}}. \end{array}$$ (25)

**Fig 7:**
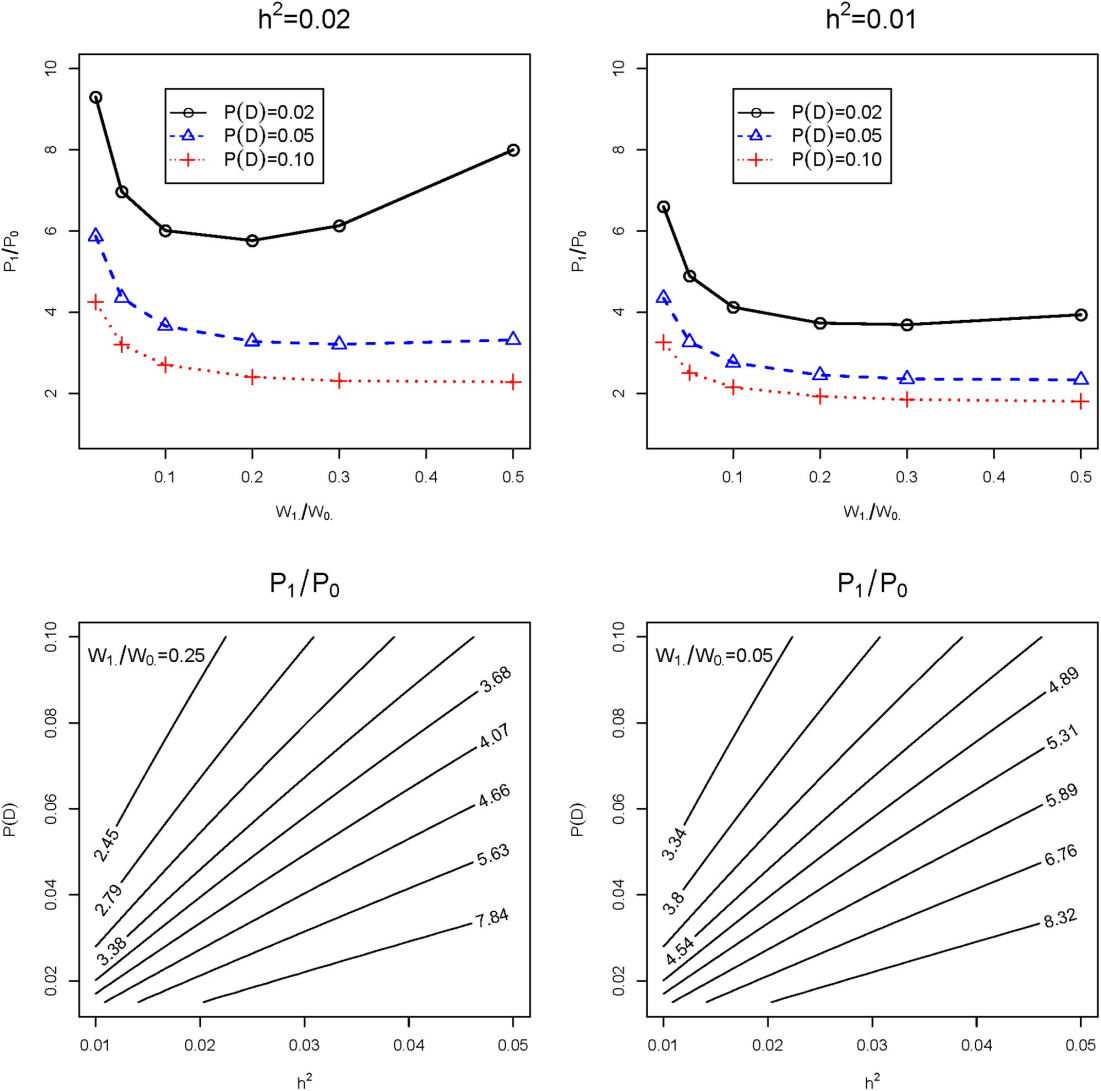
Different views of the odds, *P*_1_/*P*_0_, as a function of the ratio *W*_1⋅_/*W*_0⋅_, prevalence P(D), and heritability *h*^2^, where *P*_1_, *P*_0_ are solutions to Eqs (14) and (15). While the parameters P(D) and *h*^2^ do affect the odds *P*_1_/*P*_0_ and hence the metric Φ(*M*′, *M*), their impact is similar at different values of *W*_1⋅_/*W*_0⋅_ and hence similar for different *M*.

Fig 7 contains various views of the odds, *P*_1_/*P*_0_, as a function of the ratio *W*_1⋅_/*W*_0⋅_, prevalence P(D), and heritability *h*^2^. For any given disease model *M* with a specific ratio *W*_1⋅_/*W*_0⋅_, the odds *P*_1_/*P*_0_ is certainly affected by the choices of P(D) and *h*^2^; but these parameters also affect the odds of other disease models with different *W*_1⋅_/*W*_0⋅_-ratios in a similar manner. For example, for fixed *h*^2^, a large (and potentially wrong) choice of P(D) lowers the odds—whereas, for fixed P(D), a large (and potentially wrong) choice of *h*^2^ elevates it—for *all* disease models. As a result, even though the distances do change between different disease models and their candidate prototypes, the *relative* distances are not drastically warped. That’s why we were able to observe that the resulting prototypes were fairly robust to different choices of P(D) and *h*^2^.

We also note that the sequential screening procedure we outlined in Section 1.1 is not yet widely considered. This is understandable due to the extra computational burden—every time a pair of SNPs is added, all remaining pairs must be re-assessed. Even with today’s technology, such a procedure is still largely infeasible on the genome-wide scale. In fact, we also avoided it in our real-data analysis (Section 5) and simulation study (Section 4) for the same practical reasons, but it may deserve some attention and more systematic treatment in the future.

Finally, we note an important limitation of our current study is that we did *not* consider linkage disequilibrium (LD). It is well-known that, in GWA studies, screening algorithms can declare a specific SNP to be significant only because it is in linkage disequilibrium with another truly-associated SNP. In other words, the presence of LD can lead to false discoveries. Undoubtedly, such concerns also apply to our study here. In fact, properly accounting for LD is much more challenging and complex when we screen for epistatic effects than it is when we screen for main effects only, because the underlying question now becomes whether a *pair* of SNPs is in LD with another *pair*, rather than merely whether an individual SNP is in LD with another SNP. Thus, although studies are available for considering LD in genetic simulations [4], the complexity involved is by no means trivial on a pairwise level. We anticipate that full considerations of LD in the context of epistatis will require a considerable amount of effort in the next few years.

## Supporting information

S1 Appendix Derivation of Eq (4). (PDF)

S2 Appendix Derivation of Eq (9). (PDF)

S3 AppendixAll disease models in M.(PDF)

S4 Appendix Analysis of bipolar disorder data. GSEA results from KEGG for genes identified by MDR and RS.
(PDF)

S5 Appendix Analysis of bipolar disorder data. GSEA results from Gene Ontology and Pathway Commons for genes identified by PTY.
(PDF)

S6 Appendix A numeric example illustrating the similarity measure Φ(*M*′, *M*). (PDF)
