# Supplementary material for: Compositional epistasis detection using a few prototype disease models
Source: PLoS One. 2019 Mar 27;14(3):e0213236. doi: 10.1371/journal.pone.0213236 (PMC6436689; doi:10.1371/journal.pone.0213236)
Supplement: S1 Appendix — (PDF) [file pone.0213236.s001.pdf]

S1 Appendix
Derivation of Eq. (4)

When the SNPs are in linkage equilibrium, the population is distributed as follows.

Genotype Combination Pair 1/Pair 2	Diseased	Non-diseased
Risky/Risky	$p_{11} \sum_{i \in R} w_i \sum_{j \in R} v_j$	$(1 - p_{11}) \sum_{i \in R} w_i \sum_{j \in R} v_j$
Risky/Non-risky	$p_{10} \sum_{i \in R} w_i \sum_{j \in N} v_j$	$(1 - p_{10}) \sum_{i \in R} w_i \sum_{j \in N} v_j$
Non-risky/Risky	$p_{01} \sum_{i \in N} w_i \sum_{j \in R} v_j$	$(1 - p_{01}) \sum_{i \in N} w_i \sum_{j \in R} v_j$
Non-risky/Non-risky	$p_{00} \sum_{i \in N} w_i \sum_{j \in N} v_j$	$(1 - p_{00}) \sum_{i \in N} w_i \sum_{j \in N} v_j$

Consider the i-th genotype combination in the first pair, {A/a, B/b}. From the table above, we can see that, if it is a risky combination ($i \in R$), then its (marginal) case-control ratio is

$$r_R = \frac{p_{11} w_i \sum_{j \in R} v_j + p_{10} w_i \sum_{j \in N} v_j}{(1 - p_{11}) w_i \sum_{j \in R} v_j + (1 - p_{10}) w_i \sum_{j \in N} v_j} = \frac{p_{11} \sum_{j \in R} v_j + p_{10} \sum_{j \in N} v_j}{1 - (p_{11} \sum_{j \in R} v_j + p_{10} \sum_{j \in N} v_j)};$$

whereas if it is a non-risky combination ($i \in N$), the ratio is

$$r_N = \frac{p_{01} w_i \sum_{j \in R} v_j + p_{00} w_i \sum_{j \in N} v_j}{(1 - p_{01}) w_i \sum_{j \in R} v_j + (1 - p_{00}) w_i \sum_{j \in N} v_j} = \frac{p_{01} \sum_{j \in R} v_j + p_{00} \sum_{j \in N} v_j}{1 - (p_{01} \sum_{j \in R} v_j + p_{00} \sum_{j \in N} v_j)}.$$

Now, it is easy to see that, if Eq. (4) holds, i.e., if

$$p_{11} \sum_{j \in R} v_j + p_{10} \sum_{j \in N} v_j = p_{01} \sum_{j \in R} v_j + p_{00} \sum_{j \in N} v_j,$$

we will have $r_R = r_N$, that is, the case-control ratio will be the same for the i-th genotype combination in the first pair, regardless of whether $i \in R$ or $i \in N$.

Remark

For case-control data, both r_R and r_N would be inflated by a factor of $[1 - \mathbb{P}(D)]/\mathbb{P}(D)$, where $\mathbb{P}(D)$ is the prevalence, but this would not affect the foregoing conclusion.
