# Supplementary material for: Compositional epistasis detection using a few prototype disease models
Source: PLoS One. 2019 Mar 27;14(3):e0213236. doi: 10.1371/journal.pone.0213236 (PMC6436689; doi:10.1371/journal.pone.0213236)
Supplement: S2 Appendix — (PDF) [file pone.0213236.s002.pdf]

S2 Appendix
Derivation of Eq. (9)

Step 1

For a pair of SNPs assumed to be truly associated with the disease, suppose that M is the true, while M' is another (e.g., prototype), disease model.

Under the true model M, a randomly selected individual can be stratified into 4 different groups with probabilities given by the 2×2 table below.

	$M(G_i) = 1$	$M(G_i) = 0$	All
D	$P_1 W_{1\cdot}$	$P_0 W_{0\cdot}$	$\mathbb{P}(D)$
D^c	$(1 - P_1)W_{1\cdot}$	$(1 - P_0)W_{0\cdot}$	$1 - \mathbb{P}(D)$
All	$W_{1\cdot}$	$W_{0\cdot}$	1

Notice that

$$\begin{aligned}
\mathbb{P}(D) &= \sum_i \mathbb{P}(D|G_i)\mathbb{P}(G_i) \\
&= \sum_{M(G_i)=1} P_1 \times \mathbb{P}(G_i) + \sum_{M(G_i)=0} P_0 \times \mathbb{P}(G_i) \\
&= P_1 W_{1\cdot} + P_0 W_{0\cdot}.
\end{aligned}$$

However, under a different disease model, M', individuals would be stratified differently—in particular, according to the following table.

	$M'(G_i) = 1$	$M'(G_i) = 0$	All
D	$P_1 W_{11} + P_0 W_{01}$	$P_1 W_{10} + P_0 W_{00}$	$\mathbb{P}(D)$
D^c	$(1 - P_1)W_{11} + (1 - P_0)W_{01}$	$(1 - P_1)W_{10} + (1 - P_0)W_{00}$	$1 - \mathbb{P}(D)$
All	$W_{\cdot 1}$	$W_{\cdot 0}$	1

This is because

$$\begin{aligned}
& \mathbb{P}(D \text{ and } M' = 1) \\
= \; & \mathbb{P}(D|M' = 1) \times \mathbb{P}(M' = 1) \\
= \; & \left[\underbrace{\mathbb{P}(D|M' = 1, M = 1)}_{P_1} \mathbb{P}(M = 1) + \underbrace{\mathbb{P}(D|M' = 1, M = 0)}_{P_0} \mathbb{P}(M = 0) \right] \times \mathbb{P}(M' = 1) \\
= \; & P_1 \times \mathbb{P}(M = 1 \text{ and } M' = 1) + P_0 \times \mathbb{P}(M = 0 \text{ and } M' = 1) \\
= \; & P_1 W_{11} + P_0 W_{01},
\end{aligned}$$

where, for notational convenience, we have simply written $M' = 1$ or $M = 1$ rather than $M'(G_i) = 1$ or $M(G_i) = 1$. The other three cells in the table above can be derived in a similar fashion.

Step 2

For a case-control study, the row margins are fixed. Thus for every r case-units and 1 control-unit, respectively, the probabilities in each corresponding row of the previous table are rescaled, as shown here.

	$M'(G_i) = 1$	$M'(G_i) = 0$	All
Cases	$\dfrac{r(P_1W_{11} + P_0W_{01})}{\mathbb{P}(D)}$	$\dfrac{r(P_1W_{10} + P_0W_{00})}{\mathbb{P}(D)}$	r
Controls	$\dfrac{(1-P_1)W_{11} + (1-P_0)W_{01}}{1-\mathbb{P}(D)}$	$\dfrac{(1-P_1)W_{10} + (1-P_0)W_{00}}{1-\mathbb{P}(D)}$	1

Step 3

We can now rearrange the last table according to how the two disease models, M and M', have stratified the $r+1$ case-control units, by summing over W_{11}, W_{10}, W_{01}, and W_{00}, respectively. This gives

	$M(G_i) = 1$	$M(G_i) = 0$
$M'(G_i) = 1$	$\dfrac{rP_1W_{11}}{\mathbb{P}(D)} + \dfrac{(1-P_1)W_{11}}{1-\mathbb{P}(D)}$	$\dfrac{rP_0W_{01}}{\mathbb{P}(D)} + \dfrac{(1-P_0)W_{01}}{1-\mathbb{P}(D)}$
$M'(G_i) = 0$	$\dfrac{rP_1W_{10}}{\mathbb{P}(D)} + \dfrac{(1-P_1)W_{10}}{1-\mathbb{P}(D)}$	$\dfrac{rP_0W_{00}}{\mathbb{P}(D)} + \dfrac{(1-P_0)W_{00}}{1-\mathbb{P}(D)}$

,

which can be algebraically simplified to

	$M(G_i) = 1$	$M(G_i) = 0$
$M'(G_i) = 1$	$\dfrac{UW_{11}}{\mathbb{P}(D)[1-\mathbb{P}(D)]}$	$\dfrac{VW_{01}}{\mathbb{P}(D)[1-\mathbb{P}(D)]}$
$M'(G_i) = 0$	$\dfrac{UW_{10}}{\mathbb{P}(D)[1-\mathbb{P}(D)]}$	$\dfrac{VW_{00}}{\mathbb{P}(D)[1-\mathbb{P}(D)]}$

,

where

$$U = rP_1 + \mathbb{P}(D) - (r+1)P_1\mathbb{P}(D) = rP_1[1-\mathbb{P}(D)] + (1-P_1)\mathbb{P}(D),$$

and

$$V = rP_0 + \mathbb{P}(D) - (r+1)P_0\mathbb{P}(D) = rP_0[1-\mathbb{P}(D)] + (1-P_0)\mathbb{P}(D).$$

Calculating the Φ-coefficient of the final 2×2 contingency table above gives Eq. (9).
