# Supplementary material for: Compositional epistasis detection using a few prototype disease models
Source: PLoS One. 2019 Mar 27;14(3):e0213236. doi: 10.1371/journal.pone.0213236 (PMC6436689; doi:10.1371/journal.pone.0213236)
Supplement: S3 Appendix — (PDF) [file pone.0213236.s003.pdf]

S3 Appendix
All disease models in $\mathcal{M}$

1	0	0	0	13	0	0	0	25	0	0	1	37	0	0	1	49	0	0	0	61	0	0	1
	0	0	0		0	0	0		0	0	1		0	1	0		1	0	0		1	1	0
	0	0	1		1	0	1		1	1	1		1	1	1		0	1	1		0	0	1
2	0	0	0	14	0	0	0	26	0	0	0	38	0	0	1	50	0	0	0	62	0	0	1
	0	0	0		0	0	1		0	1	0		0	1	1		1	0	1		1	1	1
	0	1	0		1	0	0		1	0	0		1	1	0		0	1	0		0	0	0
3	0	0	0	15	0	0	0	27	0	0	0	39	0	0	1	51	0	0	0	63	0	0	1
	0	0	0		0	0	1		0	1	0		0	1	1		1	0	1		1	1	1
	0	1	1		1	0	1		1	0	1		1	1	1		0	1	1		0	0	1
4	0	0	0	16	0	0	1	28	0	0	0	40	0	0	0	52	0	0	1	64	0	0	0
	0	0	1		0	0	0		0	1	1		1	0	0		1	0	0		1	1	0
	0	1	0		1	0	0		1	0	0		0	0	0		0	1	0		0	1	0
5	0	0	0	17	0	0	1	29	0	0	0	41	0	0	0	53	0	0	1	65	0	0	0
	0	0	1		0	0	0		0	1	1		1	0	0		1	0	0		1	1	0
	0	1	1		1	0	1		1	0	1		0	0	1		0	1	1		0	1	1
6	0	0	0	18	0	0	0	30	0	0	1	42	0	0	0	54	0	0	1	66	0	0	0
	0	1	0		0	0	0		0	1	0		1	0	1		1	0	1		1	1	1
	0	0	0		1	1	0		1	0	0		0	0	0		0	1	0		0	1	0
7	0	0	0	19	0	0	0	31	0	0	1	43	0	0	0	55	0	0	1	67	0	0	0
	0	1	0		0	0	0		0	1	0		1	0	1		1	0	1		1	1	1
	0	0	1		1	1	1		1	0	1		0	0	1		0	1	1		0	1	1
8	0	0	0	20	0	0	0	32	0	0	0	44	0	0	1	56	0	0	0	68	0	0	1
	0	1	0		0	0	1		0	1	0		1	0	0		1	1	0		1	1	0
	0	1	0		1	1	0		1	1	0		0	0	0		0	0	0		0	1	0
9	0	0	0	21	0	0	0	33	0	0	0	45	0	0	1	57	0	0	0	69	0	0	1
	0	1	0		0	0	1		0	1	0		1	0	0		1	1	0		1	1	0
	0	1	1		1	1	1		1	1	1		0	0	1		0	0	1		0	1	1
10	0	0	0	22	0	0	1	34	0	0	0	46	0	0	1	58	0	0	0	70	0	0	1
	0	1	1		0	0	0		0	1	1		1	0	1		1	1	1		1	1	1
	0	1	0		1	1	0		1	1	0		0	0	0		0	0	0		0	1	0
11	0	0	0	23	0	0	1	35	0	0	0	47	0	0	1	59	0	0	0	71	0	0	1
	0	1	1		0	0	0		0	1	1		1	0	1		1	1	1		1	1	1
	0	1	1		1	1	1		1	1	1		0	0	1		0	0	1		0	1	1
12	0	0	0	24	0	0	1	36	0	0	1	48	0	0	0	60	0	0	1	72	0	1	0
	0	0	0		0	0	1		0	1	0		1	0	0		1	1	0		1	0	0
	1	0	0		1	1	0		1	1	0		0	1	0		0	0	0		0	0	0

(to be continued ...)

(... continued)

73	0 1 0<br>1 0 0<br>0 0 1	85	0 0 0<br>1 0 0<br>1 0 1	97	0 0 1<br>1 0 0<br>1 1 1	109	0 0 0<br>1 1 0<br>1 1 1	121	0 1 1<br>1 0 0<br>1 0 1	133	0 1 0<br>1 1 1<br>1 0 1
74	0 1 0<br>1 0 0<br>0 1 0	86	0 0 0<br>1 0 1<br>1 0 0	98	0 0 1<br>1 0 1<br>1 1 0	110	0 0 0<br>1 1 1<br>1 1 0	122	0 1 0<br>1 0 0<br>1 1 0	134	0 1 1<br>1 1 0<br>1 0 0
75	0 1 0<br>1 0 0<br>0 1 1	87	0 0 0<br>1 0 1<br>1 0 1	99	0 0 1<br>1 0 1<br>1 1 1	111	0 0 0<br>1 1 1<br>1 1 1	123	0 1 0<br>1 0 0<br>1 1 1	135	0 1 1<br>1 1 0<br>1 0 1
76	0 1 0<br>1 0 1<br>0 1 0	88	0 0 1<br>1 0 0<br>1 0 0	100	0 0 0<br>1 1 0<br>1 0 0	112	0 0 1<br>1 1 0<br>1 1 0	124	0 1 0<br>1 0 1<br>1 1 0	136	0 1 0<br>1 1 0<br>1 1 0
77	0 1 0<br>1 0 1<br>0 1 1	89	0 0 1<br>1 0 0<br>1 0 1	101	0 0 0<br>1 1 0<br>1 0 1	113	0 0 1<br>1 1 0<br>1 1 1	125	0 1 0<br>1 0 1<br>1 1 1	137	0 1 0<br>1 1 0<br>1 1 1
78	0 1 0<br>1 1 0<br>0 0 0	90	0 0 1<br>1 0 1<br>1 0 0	102	0 0 0<br>1 1 1<br>1 0 0	114	0 0 1<br>1 1 1<br>1 1 0	126	0 1 1<br>1 0 0<br>1 1 0	138	0 1 0<br>1 1 1<br>1 1 0
79	0 1 0<br>1 1 0<br>0 0 1	91	0 0 1<br>1 0 1<br>1 0 1	103	0 0 0<br>1 1 1<br>1 0 1	115	0 0 1<br>1 1 1<br>1 1 1	127	0 1 1<br>1 0 0<br>1 1 1	139	0 1 0<br>1 1 1<br>1 1 1
80	0 1 0<br>1 1 0<br>0 1 0	92	0 0 0<br>1 0 0<br>1 1 0	104	0 0 1<br>1 1 0<br>1 0 0	116	0 1 0<br>1 0 0<br>1 0 0	128	0 1 1<br>1 0 1<br>1 1 0	140	0 1 1<br>1 1 0<br>1 1 0
81	0 1 0<br>1 1 0<br>0 1 1	93	0 0 0<br>1 0 0<br>1 1 1	105	0 0 1<br>1 1 0<br>1 0 1	117	0 1 0<br>1 0 0<br>1 0 1	129	0 1 1<br>1 0 1<br>1 1 1	141	0 1 1<br>1 1 0<br>1 1 1
82	0 1 0<br>1 1 1<br>0 1 0	94	0 0 0<br>1 0 1<br>1 1 0	106	0 0 1<br>1 1 1<br>1 0 0	118	0 1 0<br>1 0 1<br>1 0 0	130	0 1 0<br>1 1 0<br>1 0 0	142	0 1 1<br>1 1 1<br>1 1 0
83	0 1 0<br>1 1 1<br>0 1 1	95	0 0 0<br>1 0 1<br>1 1 1	107	0 0 1<br>1 1 1<br>1 0 1	119	0 1 0<br>1 0 1<br>1 0 1	131	0 1 0<br>1 1 0<br>1 0 1	143	0 1 1<br>1 1 1<br>1 1 1
84	0 0 0<br>1 0 0<br>1 0 0	96	0 0 1<br>1 0 0<br>1 1 0	108	0 0 0<br>1 1 0<br>1 1 0	120	0 1 1<br>1 0 0<br>1 0 0	132	0 1 0<br>1 1 1<br>1 0 0		
