# Supplementary material for: Compositional epistasis detection using a few prototype disease models
Source: PLoS One. 2019 Mar 27;14(3):e0213236. doi: 10.1371/journal.pone.0213236 (PMC6436689; doi:10.1371/journal.pone.0213236)
Supplement: S4 Appendix — GSEA results from KEGG for genes identified by MDR and RS. (PDF) [file pone.0213236.s004.pdf]

S4 Appendix
GSEA results from KEGG for genes identified by MDR and RS

For comparison, Table S1 lists the statistically enriched pathways from KEGG (multiple-testing adjusted p-value < 0.05) for genes identified by MDR and Table S2, for genes identified by RS. Note that the WebGestalt tool, which we used to perform GSEA (see Section 5.3 of main text), restricts the number of overlapping genes from the candidate and reference lists to be at least 2 — i.e., both O and C must be ≥ 2 in the tables below. This explains why Table S2 is so short, and corroborates to a certain extent our finding that RS tends to produce the most inflated (nominal) measure of association (see Section 2.3 and Table 6 of main text).

Table S1: Analysis of bipolar disorder data. GSEA results from KEGG for genes identified by MDR. O = number of genes in the discovered set; C = total number of genes in the given pathway.

Line	Name	O	C	Adjusted p-value
1	oocyte meiosis	3	124	0.001
2	adrenergic signaling in cardiomyocytes	3	149	0.002
3	bile secretion	2	71	0.007
4	human T-cell leukemia virus 1 infection	3	255	0.009
5	insulin secretion	2	85	0.010
6	HTLV-I infection	3	258	0.010
7	progesterone-mediated oocyte maturation	2	98	0.013
8	cell cycle	2	124	0.020
9	phospholipase D signaling pathway	2	144	0.027

Table S2: Analysis of bipolar disorder data. GSEA results from KEGG for genes identified by RS. O = number of genes in the discovered set; C = total number of genes in the given pathway.

Line	Name	O	C	Adjusted p-value
1	oocyte meiosis	2	124	0.002
