# Supplementary material for: Compositional epistasis detection using a few prototype disease models
Source: PLoS One. 2019 Mar 27;14(3):e0213236. doi: 10.1371/journal.pone.0213236 (PMC6436689; doi:10.1371/journal.pone.0213236)
Supplement: S5 Appendix — GSEA results from Gene Ontology and Pathway Commons for genes identified by PTY. (PDF) [file pone.0213236.s005.pdf]

S5 Appendix
GSEA results from Gene Ontology and Pathway Commons for genes identified by PTY

Gene Ontology

Table S3 lists the statistically enriched pathways from Gene Ontology (multiple-testing adjusted p-value < 0.05). Quite a few of them turned out to be related to neurons and/or neuronal activities, which to some extent confirmed the relevance of the gene set we identified. For example, the pathway labelled "regulation of synaptic plasticity" (line 1) is highly relevant to both the pathophysiology and the treatment of bipolar disorder [1]. Animal models have also shown that over-strengthened and/or weakened synapses at different circuits in the brain can disturb brain functions in parallel, causing manic-like or depressive-like behaviors [2]. Similarly, the pathway labelled "neuropeptide signaling" (line 3) is integral to the modulation of membrane excitability, synaptic transmission and synaptic development [3]; while the one labelled "neuron projection" (line 11) has previously been reported to be enriched in a GWAS of bipolar disorder [4].

Table S3: Analysis of bipolar disorder data. GSEA results from Gene Ontoloty. O = number of genes in the discovered set; C = total number of genes in the given pathway.

				p-value	
Line	Name	O	C	Nominal	Adjusted
1	regulation of synaptic plasticity	4	96	0.0009	0.05
2	generation of neurons	13	1073	0.0009	0.05
3	neuropeptide signalling pathway	4	89	0.0007	0.05
4	optic nerve development	2	8	0.0006	0.05
5	organonitrogen compound biosynthetic process	18	1688	0.0004	0.05
6	oligosaccharide metabolic process	3	37	0.0006	0.05
7	neuron recognition	3	30	0.0003	0.05
8	cell recognition	4	77	0.0004	0.05
9	ion binding	38	5820	0.0004	0.04
10	axon	8	286	$\ll 0.0001$	< 0.01
11	neuron projection	11	651	$\ll 0.0001$	< 0.01

Pathway Commons

Table S4 lists the statistically enriched pathways from Pathway Commons (multiple-testing adjusted p-values < 0.1). Quite a few of them have been associated with various neural activities, neural disorders, and specifically bipolar disorder itself. For example, insulin (line 1) plays a critical role in the central nervous system, contributing to physiological processes such as neuroplasticity, neuromodulation, and neurotrophism [5]. Animal experiments have also shown that the injection of insulin in the brain increases both the amount and the activity of dopamine transporters, which may play a role in bipolar disorder [6]. The ephrin/eph signaling pathway (lines 5 and 9) affects the structure and connectivity of the dopaminergic pathway [7], and it also coordinates multiple aspects of neural development such as cell migration and axon targeting [8]. The Arf6 downstream pathway (lines 13, 14 and 18) regulates neuronal migration [9]. The N-cadherin (line 51) is important for asymmetrical cellular processes in developing neurons and for regulating neuronal polarity [10, 11]. Altered CDC42 signaling pathways (lines 53 and 54) have been observed in patients schizophrenia [12] and, finally, evidence is also emerging that the Wnt pathway (lines 61, 64 and 68) is important for bipolar disorder [13–15].

In addition, many pathways in this table (e.g., lines 4, 7, 9, 10, 11, 12, and so on) are related to cell signaling. Neurotransmitters are, of course, important signaling molecules; and so are hormones, and many

of them (e.g., glucocorticoids, thyroid hormones and gonadal steroids) are known to mediate symptoms observed in mood disorders, e.g., triggering of episodes in the postpartum period [16]. There has also been recent, specific suggestions that some signal transduction pathways may play an integral role in the pathophysiology and treatment of bipolar disorder [16].

Table S4: Analysis of bipolar disorder data. GSEA results from Pathway Commons. O = number of genes in the discovered set; C = total number of genes in the given pathway.

				p-value	
Line	Name	O	C	Nominal	Adjusted
1	Insulin Pathway	8	1288	0.0015	0.0034
2	Thrombin/protease-activated receptor (PAR) pathway	8	1300	0.0016	0.0034
3	S1P1 pathway	8	1288	0.0015	0.0034
4	IL5-mediated signaling events	8	1292	0.0015	0.0034
5	EphrinB-EPHB pathway	3	60	0.0001	0.0034
6	Signaling events mediated by focal adhesion kinase	8	1288	0.0015	0.0034
7	ErbB receptor signaling network	9	1312	0.0004	0.0034
8	IGF1 pathway	8	1291	0.0015	0.0034
9	Ephrin B reverse signaling	2	30	0.0012	0.0034
10	mTOR signaling pathway	8	1288	0.0015	0.0034
11	LKB1 signaling events	8	1308	0.0016	0.0034
12	PAR1-mediated thrombin signaling events	8	1299	0.0016	0.0034
13	Arf6 downstream pathway	8	1288	0.0015	0.0034
14	Arf6 trafficking events	8	1288	0.0015	0.0034
15	Internalization of ErbB1	8	1288	0.0015	0.0034
16	Signaling events mediated by VEGFR1 and VEGFR2	8	1296	0.0016	0.0034
17	Endothelins	8	1307	0.0016	0.0034
18	Arf6 signaling events	8	1288	0.0015	0.0034
19	PDGFR-beta signaling pathway	8	1288	0.0015	0.0034
20	Class I PI3K signaling events mediated by Akt	8	1288	0.0015	0.0034
21	Urokinase-type plasminogen activator (uPA) and uPAR-mediated signaling	8	1288	0.0015	0.0034
22	IL3-mediated signaling events	8	1295	0.0015	0.0034
23	PDGF receptor signaling network	8	1293	0.0015	0.0034
23	Metabolism	7	824	0.0005	0.0034
24	IFN-gamma pathway	8	1296	0.0016	0.0034
25	Alpha9 beta1 integrin signaling events	8	1305	0.0016	0.0034
26	Plasma membrane estrogen receptor signaling	8	1301	0.0016	0.0034
27	Metabolism of amino acids and derivatives	4	188	0.0003	0.0034
28	EGF receptor (ErbB1) signaling pathway	8	1288	0.0015	0.0034
29	ErbB1 downstream signaling	8	1288	0.0015	0.0034
30	Nectin adhesion pathway	8	1295	0.0015	0.0034
31	Amine-derived hormones	2	14	0.0003	0.0034

(continued on next page ...)

Table S4 (... continued from previous page)

Line	Name	O	C	p-value Nominal	p-value Adjusted
32	Class I PI3K signaling events	8	1288	0.0015	0.0034
33	Syndecan-1-mediated signaling events	8	1300	0.0016	0.0034
34	Glypican 1 network	8	1299	0.0016	0.0034
35	GMCSF-mediated signaling events	8	1292	0.0015	0.0034
36	VEGF and VEGFR signaling network	8	1304	0.0016	0.0034
37	Proteoglycan syndecan-mediated signaling events	9	1345	0.0004	0.0034
38	EGFR-dependent Endothelin signaling events	8	1289	0.0015	0.0034
39	Signaling events mediated by Hepatocyte Growth Factor Receptor (c-Met)	8	1293	0.0015	0.0034
40	Sphingosine 1-phosphate (S1P) pathway	8	1311	0.0017	0.0035
41	TRAIL signaling pathway	8	1328	0.0018	0.0036
42	Glypican pathway	8	1338	0.0019	0.0038
43	Beta1 integrin cell surface interactions	8	1351	0.0020	0.0039
44	EPHB forward signaling	2	40	0.0021	0.0040
45	Integrin family cell surface interactions	8	1378	0.0023	0.0043
46	AP-1 transcription factor network	5	623	0.0041	0.0074
47	Integrin-linked kinase signaling	5	656	0.0051	0.0090
48	Signaling by SCF-KIT	2	66	0.0057	0.0099
49	Axon guidance	3	219	0.0062	0.0105
50	Posttranslational regulation of adherens junction stability and dissassembly	3	231	0.0072	0.0120
51	N-cadherin signaling events	3	251	0.0090	0.0144
52	Integration of energy metabolism	2	83	0.0088	0.0144
53	CDC42 signaling events	5	757	0.0093	0.0146
54	Regulation of CDC42 activity	5	770	0.0099	0.0153
55	Stabilization and expansion of the E-cadherin adherens junction	3	275	0.0115	0.0171
56	E-cadherin signaling in the nascent adherens junction	3	275	0.0115	0.0171
57	E-cadherin signaling events	3	280	0.0121	0.0177
58	Regulation of nuclear beta catenin signaling and target gene transcription	2	135	0.0221	0.0317
59	Validated transcriptional targets of AP1 family members Fra1 and Fra2	2	136	0.0224	0.0317
60	EGFR1	2	138	0.0230	0.0320
61	Canonical Wnt signaling pathway	2	155	0.0286	0.0392
62	Regulation of p38-alpha and p38-beta	2	164	0.0317	0.0428
63	Developmental Biology	3	433	0.0373	0.0495
64	Noncanonical Wnt signaling pathway	2	182	0.0383	0.0501
65	p53 pathway	2	189	0.0410	0.0520
66	p38 MAPK signaling pathway	2	189	0.0410	0.0520

(continued on next page ...)

Table S4 (... continued from previous page)

Line	Name	O	C	p-value Nominal	p-value Adjusted
67	CXCR4-mediated signaling events	2	192	0.0422	0.0527
68	Wnt signaling network	2	200	0.0454	0.0559
69	Glypican 3 network	2	206	0.0478	0.0580
70	Syndecan-4-mediated signaling events	2	209	0.0491	0.0588
71	BMP receptor signaling	2	226	0.0564	0.0666
72	Signal Transduction	5	1231	0.0576	0.0671
73	IL1-mediated signaling events	2	234	0.0599	0.0688
74	ATR signaling pathway	2	250	0.0673	0.0763

References

1. Gisabella B, Bolshakov VY, Benes FM. Regulation of synaptic plasticity in a schizophrenia model. Proceedings of the National Academy of Sciences of the United States of America. 2005;102(37):13301–13306.

2. Du J, Quiroz JA, Gray NA, Szabo ST, Zarate Jr CA, Manji HK. Regulation of cellular plasticity and resilience by mood stabilizers: the role of AMPA receptor trafficking. Dialogues In Clinical Neuroscience. 2004;6(2):143.

3. Chen X, Ganetzky B. A neuropeptide signaling pathway regulates synaptic growth in Drosophila. The Journal of Cell Biology. 2012;196(4):529–543.

4. Forstner A, Hofmann A, Maaser A, Sumer S, Khudayberdiev S, Mühleisen T, et al. Genome-wide analysis implicates microRNAs and their target genes in the development of bipolar disorder. Translational Psychiatry. 2015;5(11):e678.

5. Brietzke E, Kapczinski F, Grassi-Oliveira R, Grande I, Vieta E, McIntyre RS. Insulin dysfunction and allostatic load in bipolar disorder. Expert Review of Neurotherapeutics. 2011;11(7):1017–1028.

6. Cousins DA, Butts K, Young AH. The role of dopamine in bipolar disorder. Bipolar Disorders. 2009;11(8):787–806.

7. Lin L, Lesnick TG, Maraganore DM, Isacson O. Axon guidance and synaptic maintenance: preclinical markers for neurodegenerative disease and therapeutics. Trends in Neurosciences. 2009;32(3):142–149.

8. Cramer KS, Miko IJ. Eph-ephrin signaling in nervous system development. F1000Research. 2016;5.

9. Hara Y, Fukaya M, Hayashi K, Kawauchi T, Nakajima K, Sakagami H. ADP ribosylation factor 6 regulates neuronal migration in the developing cerebral cortex through FIP3/Arfophilin-1-dependent endosomal trafficking of N-cadherin. Eneuro. 2016;3(4):ENEURO–0148.

10. Gärtner A, Fornasiero EF, Dotti CG. Cadherins as regulators of neuronal polarity. Cell adhesion & Migration. 2015;9(3):175–182.

11. Gärtner A, Fornasiero EF, Munck S, Vennekens K, Seuntjens E, Huttner WB, et al. N-cadherin specifies first asymmetry in developing neurons. The EMBO journal. 2012;31(8):1893–1903.

12. Ide M, Lewis DA. Altered cortical CDC42 signaling pathways in schizophrenia: implications for dendritic spine deficits. Biological Psychiatry. 2010;68(1):25–32.

13. Gould TD, Manji HK. The Wnt signaling pathway in bipolar disorder. The Neuroscientist. 2002;8(5):497–511.

14. Valvezan AJ, Klein PS. GSK-3 and Wnt signaling in neurogenesis and bipolar disorder. Frontiers in Molecular Neuroscience. 2012;5.

15. Watkins C, Sawa A, Pomper M. Glia and immune cell signaling in bipolar disorder: insights from neuropharmacology and molecular imaging to clinical application. Translational Psychiatry. 2014;4(1):e350.

16. Manji HK, Quiroz JA, Payne JL, Singh J, Lopes BP, Viegas JS, et al. The underlying neurobiology of bipolar disorder. World Psychiatry. 2003;2(3):136–146.
