# Supplementary material for: Compositional epistasis detection using a few prototype disease models
Source: PLoS One. 2019 Mar 27;14(3):e0213236. doi: 10.1371/journal.pone.0213236 (PMC6436689; doi:10.1371/journal.pone.0213236)
Supplement: S6 Appendix — (PDF) [file pone.0213236.s006.pdf]

S6 Appendix
A numeric example illustrating the similarity metric $\Phi(M', M)$

Consider a pair of SNPs, each with MAF=0.2. Suppose that the true disease model is M, and that there are two potential prototypes, M' or M'', as shown below. Which candidate is more similar to, and hence a better prototype for, M?

$\mathbb{P}(G_i)$

	AA	Aa	aa
BB	0.4096	0.2048	0.0256
Bb	0.2048	0.1024	0.0128
bb	0.0256	0.0128	0.0016

M

	AA	Aa	aa
BB	0	0	0
Bb	0	1	1
bb	0	1	1

M'

	AA	Aa	aa
BB	0	0	**1**
Bb	0	1	1
bb	**1**	1	**0**

M''

	AA	Aa	aa
BB	0	0	0
Bb	0	1	**0**
bb	0	**0**	**0**

Here, model M' differs from M on the genotypes, aaBB, AAbb and aabb, with

$$W'_{11} = 0.1280, \quad W'_{10} = 0.0016, \quad W'_{01} = 0.0512, \quad W'_{00} = 0.8192;$$

and model M'' differs from M on the genotypes, aaBb, Aabb and aabb, with

$$W''_{11} = 0.1024, \quad W''_{10} = 0.0272, \quad W''_{01} = 0.0000, \quad W''_{00} = 0.8704.$$

The entries for which $M' \neq M$ are emboldened in the table above, and so are those for which $M'' \neq M$.

Overall, M'' has less disagreement with M than does M', as can be seen from the inequality:

$$\sum_{k \neq \ell} W''_{k\ell} = (0.0272 + 0.0000) < (0.0016 + 0.0512) = \sum_{k \neq \ell} W'_{k\ell}.$$

However, while M' has more disagreement with M overall, it actually has more agreement with M than does M'' on the set $\mathcal{G}_1 \equiv \{G_i : M(G_i) = 1\}$—i.e., not only AaBb but also aaBb and Aabb, although it also has additional disagreements with M on the set $\mathcal{G}_0 \equiv \{G_i : M(G_i) = 0\}$—i.e., aaBB, AAbb, whereas M'' agrees with M completely on $\mathcal{G}_0$.

The similarity metric Φ—Eq. (9) of main text—is acutely sensitive to such a difference. On population data ($r = r_0$; see Section 6 of main text), we have

$$\frac{U}{V} = 1 \quad \Rightarrow \quad \Phi(M', M) = 0.273 \quad \text{and} \quad \Phi(M'', M) = 0.294,$$

so M'' would be considered more similar to, and hence a better prototype for, M. However, on case-control data ($r \gg r_0$), the ratio $U/V \approx P_1/P_0 > 1$; see, again, Section 6 of main text. As a concrete example here, suppose $U/V \approx P_1/P_0 = 5$, which is a fairly typical value in practice; then, we get

$$\frac{U}{V} = 5 \quad \Rightarrow \quad \Phi(M', M) = 0.310 \quad \text{and} \quad \Phi(M'', M) = 0.278,$$

so M' would be considered a better prototype for M instead of M''. We think this is a desirable property of our similarity metric Φ, in that it is more important for a prototype candidate M' to agree with M on the set $\mathcal{G}_1$ than on $\mathcal{G}_0$.
